# Aloe-emodin as drug candidate for cancer therapy

**DOI:** 10.18632/oncotarget.24880

**Published:** 2018-04-03

**Authors:** Nadire Özenver, Mohamed Saeed, Lütfiye Ömur Demirezer, Thomas Efferth

**Affiliations:** ^1^ Department of Pharmacognosy, Faculty of Pharmacy, Hacettepe University, 06100 Ankara, Turkey; ^2^ Department of Pharmaceutical Biology, Institute of Pharmacy and Biochemistry, Johannes Gutenberg University, 55128 Mainz, Germany

**Keywords:** Aloe-emodin, *Rumex acetosella*, cancer, multi-drug resistance, anthraquinone

## Abstract

As a leading cause of global mortality, cancer frequently cannot be cured due to the development of drug resistance. Therefore, novel drugs are required. Naturally occurring anthraquinones are mostly present in *Rumex* and *Rhamnus* species and are of interest because of their structural similarity to anthracyclines as well established anticancer drugs. In the present study, we focused on the structural elucidation of phytochemicals from *R. acetosella* as well as the investigation of cytotoxicity and modes of action of the main anthraquinone aglycons (emodin, Aloe-emodin, physcion, rhein). Resazurin reduction and protease viability marker assays were conducted to test their cytotoxicity. Microarray-based gene expression profiling was performed to identify cellular pathways affected by the compounds, which was validated by qPCR analyses and functional assays. Flow cytometry was used to measure cell cycle distribution, apoptosis and necrosis, induction of reactive oxygen species (ROS) and disruption of mitochondrial membrane potential (MMP). The comet assay was used to detect DNA damage. Aloe-emodin as the most cytotoxic compound revealed IC_50_ values from 9.872 μM to 22.3 μM in drug-sensitive wild-type cell lines and from 11.19 μM to 33.76 μM in drug-resistant sublines, was selected to investigate its mechanism against cancer. Aloe-emodin-induced S phase arrest, ROS generation, DNA damage and apoptosis. Microarray hybridization revealed a profile of deregulated genes in Aloe-emodin-treated CCRF-CEM cells with diverse functions such as cell death and survival, cellular growth and proliferation, cellular development, gene expression, cellular function and maintenance. Aloe-emodin as well as *R. acetosella* deserve further investigations as possible antineoplastic drug candidates.

## INTRODUCTION

Cancer is one of the leading cause of death worldwide. In 2012, 14.1 Mio people were diagnosed with cancer [1] and new cases with cancer is predicted to ascend by 70% over the next 20 years [2].

Despite notable improvements in cancer research in the past few decades, many cancer patients still cannot be cured due to the development of drug resistance. Even worse, tumors frequently develop resistance not only to single drugs, but also towards many drugs at the same time. This phenomenon was termed multidrug resistance and decreases the success of chemotherapy [3]. The response of tumor cells to cytotoxic agents is frequently determined by multiple factors [4, 5].

Natural sources might have importance as potential drug candidates. Evidently, 69% of anticancer drugs approved between the 1980s and 2002 were either natural products or developed based on knowledge gained from natural substances [6]. Natural compounds are indispensable not only as chemically established anticancer drugs (*e.g*. anthracyclines, *Vinca* alkaloids, taxanes, camptothecins etc.), but also as lead compounds for the development of novel targeted chemotherapeutics with improved antitumor efficacy and fewer side effects [7].

Among the various chemical classes of natural products, anthraquinones are characterized by their large structural diversity, pronounced biological activity and low toxicity [8]. Anthraquinones are mostly present in the families of Fabaceae (*Cassia*), Liliaceae (*Aloe*), Polygonaceae (*Rumex*), Rhamnaceae (*Rhamnus*), Rubiaceae (*Asperula*, *Gallium*,*Rubia*), and Scrophulariaceae [9]. Anthraquinones inhibited growth of tumor cell lines derived from breast [10–12], lung [11], cervix [13], prostate [14], colon, central nervous system as well as of glioma [11, 15], hepatoma [16] and leukemia [17, 18]. Furthermore, the structural similarity of anthraquinone aglycons to anthracyclines as well established anticancer drugs allows to speculate on their possible activities against cancer.

*Rumex acetosella L*. (Polygonaceae) has a long tradition in folk medicine for cancer treatment [19–21], *e.g.* as component of Essiac tea in Canada [22]. The traditional use of *R. acetosella* extracts was substantiated by *in vitro* studies [23]. The plant contains anthraquinones, flavonoids and other phenolics [21, 24, 25, 26]. The constituents of the plant may account for its cytotoxicity.

In the present investigation, we focused on the cytotoxic effects of the main anthraquinone aglycons (emodin, Aloe-emodin, physcion, rhein) against cancer cells. The aims of the present study were:

(1) The structural elucidation of phytochemicals from *R. acetosella* and

(2) The identification of cellular and molecular factors determining cytotoxicity and acquired resistance. Aloe-emodin was selected from the panel of compounds in *R. acetosella.* Upon treatment of cells with Aloe-emodin, microarray-based expression profiles, reactive oxygen species (ROS), DNA damage, cell cycle arrest, as well as apoptosis and necrosis were investigated.

(3) Since tumor cells can be unresponsive to cytotoxic compounds, even if they have never been exposed to drugs before, we also studied factors of inherent resistance to Aloe-emodin by microarray-based mRNA profiling.

## RESULTS

### Structural determination of the isolated phytochemicals

**Figure 1:**
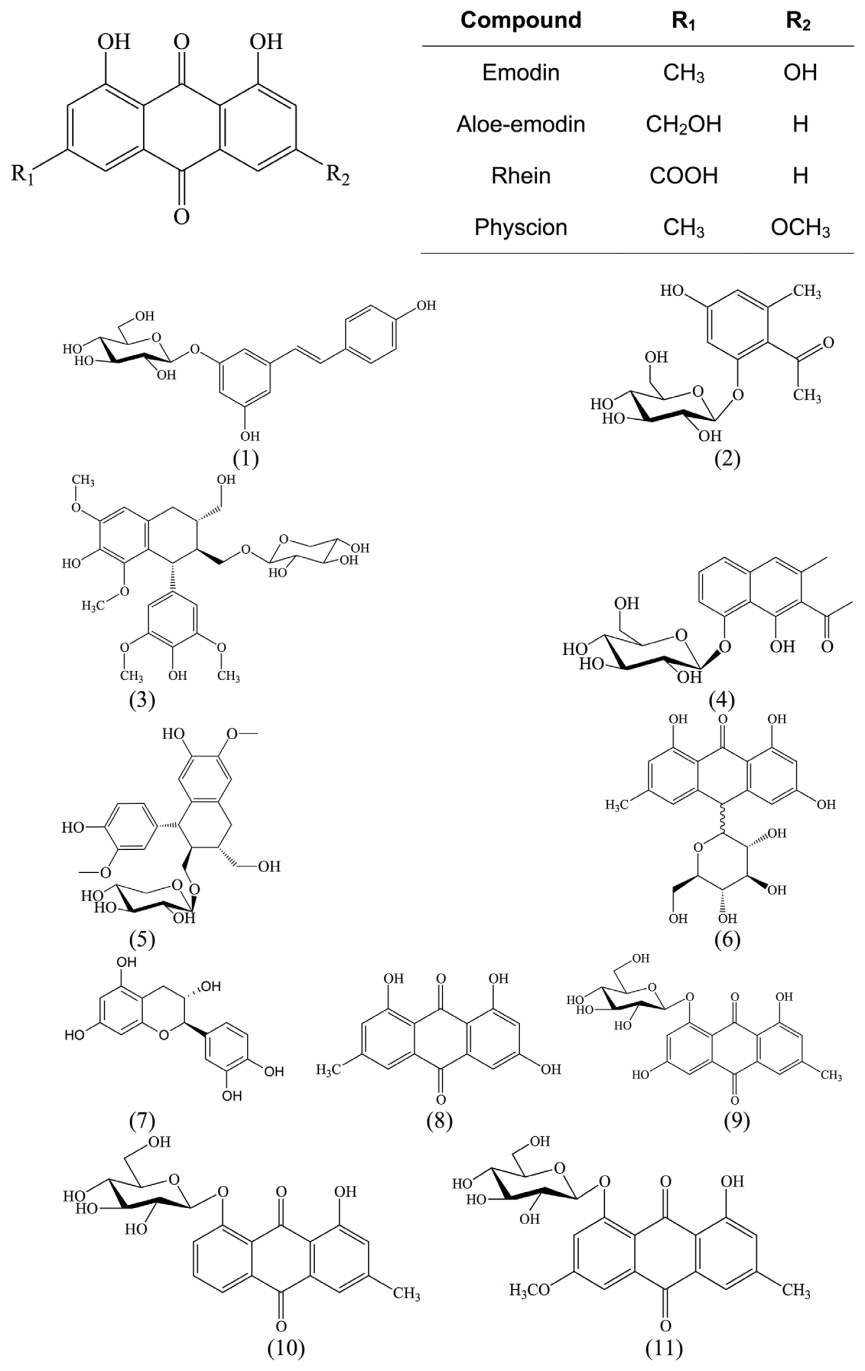
Chemical structures of main anthraquinone aglycons and isolated compounds from *R. acetosella L*

The structures of isolated compounds were elucidated with spectroscopic data. The compounds belonged to the phytochemical classes of anthraquinones, naphthalenes, stilbenoids, lignins, ethanones or tannins (Figure 1).

In addition to known substances, we isolated a new glycoside (compound 2) from the roots of *R. acetosella* and named it acetoselloside.

**Compound 1**: (E)-Piceid.

^1^H NMR (600 MHz, DMSO-*d*_6_) δ 9.56 (s, 1H, C-4‘‘-OH), 9.49 (s, 1H, C-3-OH), 7.42 – 7.37 (m, 2H, H-2‘‘, H-6‘‘), 7.03 (d, *J* = 16.3 Hz, 1H, H-2‘), 6.86 (d, *J* = 16.3 Hz, 1H, H-1‘), 6.78 – 6.73 (m, 2H, H-3‘‘, H-5‘‘), 6.72 (t, *J* = 1.8 Hz, 1H, H-6), 6.55 (t, *J* = 1.8 Hz, 1H, H-4), 6.32 (t, *J* = 1.8 Hz, 1H, H-2), 5.29 (d, *J* = 4.8 Hz, 1H, C-2‘‘‘-OH), 5.11 (s, 1H, C-3‘‘‘-OH), 5.04 (s, 1H, C-4‘‘‘-OH), 4.79 (d, *J* = 7.7 Hz, 1H, H-1‘‘‘), 4.64 (t, *J* = 5.8 Hz, 1H, C-6‘‘‘-OH), 3.72 (ddd, *J* = 11.8, 5.8, 2.1 Hz, 1H, H-6‘‘‘), 3.48 (dt, *J* = 11.8, 5.8 Hz, 1H, H-6‘‘‘), 3.31 (ddd, *J* = 9.4, 5.8, 2.1 Hz, 1H, H-5‘‘‘), 3.26 (t, *J* = 8.9 Hz, 1H, H-3‘‘‘), 3.20 (td, *J* = 8.4, 4.8 Hz, 1H, H-2‘‘‘), 3.15 (t, *J* = 9.4 Hz, 1H, H-4‘‘‘).

^13^C NMR (151 MHz, DMSO) δ 158.9 (C-1), 158.4 (C-3), 157.4 (C-4‘‘), 139.4 (C-5), 128.6 (C-2‘), 128.0 (C-2‘‘, C-6‘‘), 125.2 (C-1‘), 115.6 (C-3‘‘, C-5‘‘), 107.2 (C-4), 104.7 (C-6), 102.7 (C-2), 100.7 (C-1‘‘‘), 77.2 (C-5‘‘‘), 76.7 (C-3‘‘‘), 73.3 (C-2‘‘‘), 69.8 (C-4‘‘‘), 60.7 (C-6‘‘‘).

[α]_D_^22^ = –39.3° (MeOH, c = 0.54).

IR (ATR) ν (cm^–1^) = 3533 – 3050, 2928, 1620, 1513, 1433, 1173, 1076, 1023, 944.

HRMS (ESI) *m/z*:[M+Na]^+^ Calculated for C_20_H_22_O_8_Na 413.1212; Found: 413.1213.

**Compound 2:** Ethanone, 1-[2-(*β*-glucopyranosyloxy)-4-hydroxy-6-methylphenyl)].

^1^H NMR (600 MHz, DMSO-*d*_6_) δ 6.42 (d, *J* = 2.0 Hz, 1H, H-3), 6.27 (d, *J* = 2.0 Hz, 1H, H-5), 5.29 (d, *J* = 5.4 Hz, 1H, C-2‘-OH), 5.11 (s, 1H, C-3‘-OH), 5.05 (s, 1H, C-4‘-OH), 4.83 (d, *J* = 7.7 Hz, 1H, H-1‘), 4.56 (s, 1H, C-6‘-OH), 3.68 (dd, *J* = 11.9, 2.1 Hz, 1H, H-6‘), 3.48 (dd, *J* = 11.9, 5.4 Hz, 1H, H-6‘), 3.31 – 3.23 (m, 2H, H-3‘, H-5‘), 3.23 – 3.15 (m, 2H, H-2‘, H-4‘), 2.43 (s, 3H, CO-*Me*), 2.08 (s, 3H, C-6-*Me* ).

^13^C NMR (151 MHz, DMSO) δ 203.4 (C=O), 159.2 (C-4), 156.4 (C-2), 137.1 (C-6), 122.5 (C-1), 111.0 (C-5), 100.5 (C-1‘), 99.9 (C-3), 77.1 (C-5‘), 76.8 (C-3‘), 73.3 (C-2‘), 69.5 (C-4‘), 60.6 (C-6‘), 32.7 (CO-*Me)*, 19.6 (C-6-*Me*).

[α]_D_^22^ = –35.6° (MeOH, c = 0.46).

IR (ATR) ν (cm^–1^) = 3650 – 3000, 2928, 1654, 1605, 1464, 1328, 1258, 1173, 1077.

HRMS (ESI) *m/z*:[M+Na]^+^ Calculated for C_15_H_20_O_8_Na 351.1056; Found: 351.1047.

**Compound 3:** Lyoniside (major diastereomere being contaminated with a minor diastereomere).

^1^H NMR (600 MHz, DMSO-*d*_6_) δ 8.18 (s, 1H), 8.02 (s, 1H), 6.54 (s, 0H), 6.53 (s, 1H, H-8), 6.32 (s, 2H, H-2‘, H-6‘), 6.28 (s, 1H), 5.20 (d, *J* = 4.9 Hz, 1H, C-2‘‘-OH), 5.06 (d, *J* = 4.3 Hz, 1H, C-4‘‘-OH), 5.04 (s, 0H), 5.00 (d, *J* = 4.9 Hz, 1H, C-3‘‘-OH), 4.48 (t, *J* = 5.1 Hz, 1H, CH_2_-O*H*), 4.45 (t, *J* = 5.1 Hz, 0H), 4.25 (d, *J* = 6.6 Hz, 1H, H-4), 4.14 (d, *J* = 6.2 Hz, 0H), 4.10 (d, *J* = 7.7 Hz, 1H, H-1‘‘), 4.05 (d, *J* = 7.6 Hz, 0H), 3.76 (s, 1H), 3.76 (s, 3H, C-7-O*Me*), 3.73 – 3.64 (m, 2H, H-5‘‘, H-3α), 3.63 (s, 6H, C-3‘-O*Me*, C-5‘-O*Me*), 3.52 – 3.44 (m, 1H, H-2α), 3.35 (m (water signal), 1H, H-2α), 3.30 – 3.24 (m, 1H, H-3‘‘), 3.24 – 3.23 (m, 1H, H-3α), 3.22 (s, 3H, C-5-O*Me*), 3.13 – 3.08 (m, 1H, H-4‘‘), 3.04 – 2.98 (m, 2H, H-2‘‘, H-5‘‘), 2.65 – 2.57 (m, 1H, H-1), 2.54 – 2.51 (m, 1H, H-1), 1.94 – 1.86 (m, 1H, H-3), 1.50 (dt, *J* = 11.4, 7.7 Hz, 1H, H-2).

^13^C NMR (151 MHz, DMSO) δ 147.50 (C-3‘, C-5‘), 146.94, 146.90 (C-7), 146.52 (C-5), 146.40, 137.69, 137.63 (C-1‘), 137.27 (C-6), 137.24, 133.33, 133.24 (C-4‘), 128.45, 128.34 (C-8a), 124.94 (C-4a), 124.79, 106.65 (C-8), 105.86 (C-2‘, C-6‘), 104.10 (C-1‘‘), 103.79, 76.85 (C-4‘‘), 76.73, 73.31 (C-2‘‘), 69.61 (C-3‘‘), 68.86 (C-3α), 65.86, 65.78 (C-5’’), 63.94, 63.65 (C-2α), 58.79, 58.62 (C-5-O*Me*), 56.07 (C-3‘-O*Me*, C-5‘-O*Me*), 56.02, 55.67, 55.65 (C-7-O*Me*), 44.65 (C-3), 44.55, 41.03 (C-4), 40.95, 38.81 (C-2), 32.67 (C-1), 32.49.

[α]_D_^22^ = +3.6° (MeOH, c = 0.47).

IR (ATR) ν (cm^–1^) = 3550 –3050, 2934, 2851, 1611, 1515, 1462, 1324, 1221, 1113, 1047, 1025, 994.

HRMS (ESI) *m/z*:[M+Na]^+^ Calculated for C_27_H_36_O_12_Na 575.2104; Found: 575.2093.

**Compound 4:** Musizin-/nepodin-8-*O*-*β*-glucopyranoside.

^1^H NMR (600 MHz, Methanol-*d*_4_) δ 7.40 (dd, *J* = 8.1, 1.2 Hz, 1H, H-5), 7.35 (t, *J* = 7.9 Hz, 1H, H-6), 7.31 (dd, *J* = 7.7, 1.2 Hz, 1H, H-7), 7.13 (d, *J* = 1.1 Hz, 1H, H-4), 5.10 (d, *J* = 7.9 Hz, 1H, H-1‘), 3.93 (dd, *J* = 12.1, 2.3 Hz, 1H, H-6‘), 3.72 (dd, *J* = 12.1, 5.9 Hz, 1H, H-6‘), 3.54 (dd, *J* = 9.2, 7.9 Hz, 1H, H-2’), 3.52 – 3.49 (m, 1H, H-5‘), 3.47 (t, *J* = 9.0 Hz, 1H, H-3‘), 3.40 (dd, *J* = 9.7, 8.8 Hz, 1H, H-4‘), 2.28 (d, *J* = 0.9 Hz, 3H, C-3-*Me*).

^13^C NMR (151 MHz, MeOD) δ 208.37 (*C*=O), 156.18 (C-8), 152.93 (C-1), 138.11 (C-4a), 134.67 (C-3), 128.58 (C-6), 126.09 (C-2), 123.87 (C-5), 120.93 (C-4), 114.95 (C-8a), 111.86 (C-7), 104.34 (C-1’), 78.86 (C-5’), 78.17 (C-3’), 74.99 (C-2’), 71.29 (C-4’), 62.48 (C-6’), 31.81 (CO-*Me*), 19.91 (C-3-*Me*).

[α]_D_^22^ = –53.5° (EtOH, c = 0.26).

IR (ATR) ν (cm^–1^) = 3385, 3298, 2964, 2925, 1670, 1578, 1354, 1079.

HRMS (ESI) *m/z*:[M+Na]^+^ Calculated for C_19_H_22_O_8_Na 401.1212; Found: 401.1201.

**Compound 5:** (+)-Isolariciresinol-9-*O-β*-xylopyranoside.

^1^H NMR (600 MHz, DMSO-*d*_6_) δ 8.74 (s, 1H, C-4’-OH), 8.44 (s, 1H, C-7-OH), 6.79 (d, *J* = 2.0 Hz, 1H, H-2’), 6.68 (d, *J* = 8.0 Hz, 1H, H-5’), 6.59 (s, 1H, H-5), 6.47 (dd, *J* = 8.1, 1.9 Hz, 1H, H-6’), 6.06 (s, 1H, H-8), 5.24 (d, *J* = 4.7 Hz, 1H, C-2’’-OH), 4.99 (d, *J* = 4.8 Hz, 1H, C-3’’-OH), 4.95 (d, *J* = 5.0 Hz, 1H, C-4’’-OH), 4.41 (t, *J* = 5.1 Hz, 1H, C-3α-OH), 4.02 (d, *J* = 10.9 Hz, 1H, H-1), 3.90 (d, *J* = 7.6 Hz, 1H, H-1’’), 3.84 (dd, *J* = 9.6, 2.4 Hz, 1H, H-2α), 3.71 (s, 3H, C-3’-O*Me*), 3.70 (s, 3H, C-6-O*Me*), 3.64 (dd, *J* = 11.3, 5.4 Hz, 1H, H-5’’), 3.57 (dt, *J* = 10.5, 4.2 Hz, 1H, H-3α), 3.46 (dt, *J* = 11.0, 5.9 Hz, 1H, H-3α), 3.26 (ddt, *J* = 10.4, 8.8, 5.2 Hz, 1H, H-4’’), 3.07 (td, *J* = 8.8, 4.8 Hz, 1H, H-3’’), 3.00 – 2.94 (m, 3H, H-2α, H-2’’, H-5’’), 2.73 – 2.70 (m, 2H, H-4), 1.86 (ddq, *J* = 13.1, 6.2, 3.5 Hz, 1H, H-3), 1.68 (tt, *J* = 10.7, 2.7 Hz, 1H, H-2).

^13^C NMR (151 MHz, DMSO) δ 147.14 (C-3’), 145.50 (C-6), 144.47 (C-4’), 144.03 (C-7), 136.91 (C-1’), 132.62 (C-9/8a), 127.02 (C-10/4a), 121.05 (C-6’), 116.27 (C-8), 115.48 (C-5’), 113.86 (C-2’), 111.76 (C-5), 104.59 (C-1’’), 76.59 (C-3’’), 73.37 (C-2’’), 69.58 (C-4’’), 67.23 (C-2α), 65.70 (C-5’’), 62.60 (C-3α), 55.57 (C-6-O*Me*), 55.48 (C-3’-O*Me*), 45.64 (C-1), 44.11 (C-2), 37.56 (C-3), 32.63 (C-4).

[α]_D_^22^ = +16.9° (MeOH, c = 0.19).

HRMS (ESI) *m/z*:[M+Na]^+^ Calculated for C_25_H_32_O_10_Na 515.1893; Found: 515.1904.

**Compound 6:** Rumejaposide G/H diastereomeric mixture.

^1^H NMR (600 MHz, DMSO-*d*_6_) δ 12.17 (s, 1H, C-1-OH, A), 12.16 (s, 1H, C-1-OH-B), 12.00 (s, 1H, C-8-OH, A), 12.00 (s, 1H, C-8-OH, B), 10.74 (s, 2H, C-3-OH, both), 6.88 (d, *J* = 1.4 Hz, 1H, H-5, A), 6.84(s, 1H, H-5, B), 6.68 (s, 1H, H-7, A), 6.67 (s, 1H, H-7, B), 6.52 (d, *J* = 2.2 Hz, 1H, H-4, B), 6.47 (d, *J* = 2.3 Hz, 1H, H-4, A), 6.22 (d, *J* = 2.3 Hz, 1H, H-2. A), 6.20 (d, *J* = 2.2 Hz, 1H, H-2, B), 5.19 (d, *J* = 5.9 Hz, 1H, C-2’-OH, A), 5.14 (d, *J* = 5.9 Hz, 1H, C-2’-OH, B), 4.94 (d, *J* = 5.0 Hz, 1H, C-3’-OH, B), 4.92 (d, *J* = 4.9 Hz, 1H, C-3’-OH, A), 4.79 – 4.76 (m, 2H, C-4’-OH, both), 4.42 (d, *J* = 2.1 Hz, 1H, H-10, B), 4.41 (d, *J* = 2.1 Hz, 1H, H-10, A), 3.89 (pseudo-q, *J* = 5.5 Hz, 2H, C-6’-OH, both), 3.42 – 3.36 (m, 1H, H-6’, both), 3.27 (dd, *J* = 9.2, 2.2 Hz, 1H, H-1’, A), 3.26 (dd, *J* = 9.2, 2.2 Hz, 1H, H-1’, B), 3.19 – 3.13 (m, 2H, H-6’, both), 3.13 – 3.06 (m, 2H, H-3’, both), 2.92 (td, *J* = 9.2, 5.9 Hz, 1H, H-2’, B), 2.84 (td, *J* = 9.2, 5.9 Hz, 1H, H-2’, A), 2.79 (ddd, *J* = 9.8, 5.8, 2.2 Hz, 1H, H-5’, A), 2.74 – 2.68 (m, 3H, H-4’ both, H-5’ B), 2.34 (s, 3H, C-6-*Me*, A), 2.33 (s, 3H, C-6-*Me*, B).

^13^C NMR (151 MHz, DMSO) δ 191.86 (C=O, both), 164.84 (C-3, A), 164.32 (C-3, B), 164.15 (C-1, A), 164.07 (C-1, B), 161.32 (C-8, A), 161.12 (C-8, B), 148.51 (C-5a, A), 146.89 (C-6, B), 146.11 (C-6, A), 145.84 (C-5a, B), 144.61 (C-4a, B), 142.06 (C-4a, A), 121.58 (C-5, A), 120.15 (C-5, B), 116.20 (C-7, A), 115.97 (C-7, B), 115.18 (C-8a, A), 115.06 (C-8a, B), 110.74 (C-9a, B), 110.54 (C-9a, A), 109.71 (C-4, B), 107.96 (C-4, A), 101.64 (C-2, A), 101.55 (C-2, B), 86.33 (C-1’, A), 86.15 (C-1’, B), 81.38 (C-5’, B), 81.28 (C-5’, A), 78.68 (C-3’, B), 78.64 (C-3’, A), 70.74 (C-4’, B), 70.71 (C-4’, A), 70.53 (C-2’, A), 70.31 (C-2’, B), 61.87 (C-6’, B), 61.78 (C-6’, A), 44.44 (C-10, A), 44.16 (C-10, B), 22.12 (C-6-*Me*, A), 22.10 (C-6-*Me*, B).

[α]_D_^22^ = –3.2° (MeOH, c = 0.22).

IR (ATR) ν (cm^–1^) = 3550 – 3050, 1620, 1481, 1368, 1284, 1150, 1025, 992.

HRMS (ESI) *m/z*:[M+Na]^+^ Calculated for C_21_H_22_O_9_Na 441.1162; Found: 441.1169.

**Compound 7:** (–)-Catechin/flavan-3-ol.

^1^H NMR (600 MHz, DMSO-*d*_6_) δ 9.14 (s, 1H, Ar-OH), 8.92 (s, 2H, 2x Ar-OH), 8.85 (s, 1H, Ar-OH), 6.88 (d, *J* = 1.9 Hz, 1H, H-2‘), 6.66 (d, *J* = 8.1 Hz, 1H, H-5‘), 6.63 (dd, *J* = 8.1, 1.9 Hz, 1H, H-6‘), 5.89 (d, *J* = 2.3 Hz, 1H, H-6), 5.71 (d, *J* = 2.3 Hz, 1H, H-8), 4.72 (s, 1H, H-2), 4.68 (s, 1H, C-3-OH), 3.99 (s, 1H, H-3), 2.67 (dd, *J* = 16.3, 4.5 Hz, 1H, H-4), 2.49 – 2.45 (m, 1H, H-4).

^13^C NMR (151 MHz, DMSO) δ 156.56 (C-7), 156.25 (C-5), 155.79 (C-8a), 144.55 (C-4’), 144.52 (C-3’), 130.58 (C-1’), 117.93 (C-6’), 114.90 (C-2’), 114.76 (C-5’), 98.47 (C-4a), 95.05 (C-6), 94.06 (C-8), 78.08 (C-2), 64.93 (C-3), 28.26 (C-4).

[α]_D_^22^ = –12.6° (MeOH, c = 0.32).

IR (ATR) ν (cm^–1^) = 3571 – 3050, 2965, 1626, 1605, 1468, 1284, 1147, 1024, 911.

MS (ESI) *m/z*: [M+H]^+^ 291.1.

**Compound 8:** Emodin.

^1^H NMR (600 MHz, DMSO-*d*_6_) δ 13.15 (s, 1H, OH), 12.41 (s, 1H, OH), 7.36 (s, 1H, H-4), 7.00 (s, 1H, H-2), 6.52 (d, *J* = 2.3 Hz, 1H, H-5), 5.62 (d, *J* = 2.3 Hz, 1H, H-7), 2.35 (s, 3H, C-6-*Me*).

^13^C NMR (151 MHz, DMSO) δ 184.48 (C-10), 181.52 (C-9), 166.55, 166.44 (C-6/C-8), 161.22 (C-1), 144.93 (C-3), 134.35 (C-11), 133.37 (C-14), 123.90 (C-2), 119.40 (C-4/C-5), 115.34 (C-13), 101.64 (C-12), 107.92 (C-7), 21.76 (C-3-Me).

IR (ATR) ν (cm^–1^) = 1683, 1653, 1478, 1273, 758

MS (ESI) *m/z*: [M+H]^+^ 271.2.

**Compound 9:** Emodin-8-*O*-*β*-glucopyranoside.

^1^H NMR (600 MHz, DMSO-*d*_6_) δ 7.31 (d, *J* = 1.6 Hz, 1H, H-4), 6.98 (d, *J* = 1.6 Hz, 1H, H-2), 6.59 (d, *J* = 2.4 Hz, 1H, H-5), 6.20 (d, *J* = 2.4 Hz, 1H, H-7), 4.66 (d, *J* = 7.1 Hz, 1H, H-1’), 3.73 (d, *J* = 12.1 Hz, 1H, H-6’a), 3.50 (d, *J* = 1.6 Hz, 1H, H-6’b), 3.34 – 3.25 (m, H_2_O signal, H-2’, H-3’, H-5’), 3.19 (t, *J* = 8.8 Hz, 1H, H-4’), 2.34 (s, 3H, C-3-*Me*).

^13^C NMR (151 MHz, DMSO) δ 184.73 (C-10), 180.12 (C-9), 163.94 (C-6 and C-8), 161.76 (C-1), 144.14 (C-3), 135.62 (C-5a), 132.49 (C-4a), 123.69 (C-2), 118.23 (C-5), 118.07 (C-4), 115.76 (C-1a), 111.97 (C-7), 103.69 (C-1’), 103.53 (C-8a), 77.58 (C-5’), 75.62 (C-2’), 73.57 (C-3’), 69.79 (C-4’), 60.79 (C-6’), 21.36 (C-3-*Me*).

^1^H NMR (600 MHz, Methanol-*d*_4_) δ 7.47 (d, *J* = 1.7 Hz, 1H, H-4), 7.14 (d, *J* = 2.5 Hz, 1H, H-5), 7.02 – 7.00 (m, 1H, H-2), 6.78 (d, *J* = 2.5 Hz, 1H, H-7), 3.94 (dd, *J* = 12.2, 1.6 Hz, 1H, H-6‘a), 3.80 (dd, *J* = 12.2, 4.2 Hz, 1H, H-6‘b), 3.63 – 3.59 (m, 1H, H-2‘), 3.55 – 3.45 (m, 3H, H-3‘, H-4‘, H-5‘), 2.39 (s, 3H, C-3-*Me*).

^13^C NMR (151 MHz, MeOD) δ 185.38 (C-10), 178.72 (C-9), 164.32 (C-6 and C-8), 163.48 (C-1), 147.36 (C-3), 137.83 (C-10a), 134.37 (C-4a), 125.00 (C-2), 120.31 (C-4), 116.77 (C-5), 116.59 (C-9a), 113.94 (C-7), 110.08 (C-8a), 104.80 (C-1’), 78.60 (C-5’), 77.28 (C-3’), 74.81 (C-2’), 71.02 (C-4’), 62.29 (C-6’), 21.82 (C-3-*Me*).

[α]_D_^22^ = not possible due to high absorption.

IR (ATR) ν (cm^–1^) = 3550 – 3050, 1629, 1480, 1366, 1266, 1074, 1025, 991.

**Compounds 10 and 11:** Mixture of chrysophanol-8-*O*-*β*-glucopyranoside and physcion-8-*O*-*β*-glucopyranoside.

^1^H NMR (600 MHz, DMSO-*d*_6_) δ 7.85 (d, *J* = 8.0 Hz, 1H, H-5, A), 7.81 (t,*J* = 8.0 Hz, 1H, H-6, A), 7.67 (d, *J* = 8.0 Hz, 1H, H-7, A), 7.35 (d, *J* = 2.5 Hz, 1H, H-5, B), 7.17 (d, *J* = 2.5 Hz, 1H, H-7, B), 7.44/7.38/7.15/7.11 (broad signals which might be H-5 and H-7), 5.16 (d, *J* = 7.7 Hz, 1H, H-1’, B), 5.12 (d, *J* = 7.7 Hz, 1H, H-1’, A), 3.95 (s, 3H, C-6-O*Me*, B), 3.73 – 3.68 (m, 2H, H-6’, H-6’, A and B), 3.50 (t, *J* = 6.0 Hz, 2H, H-6’, H-6’, A and B), 3.49 – 3.31 (m, 6H, water, H-2’, H-2’, H-3’, H-3’, H-5’, H-5’, A and B), 3.22 (t, *J* = 9.3 Hz, 1H, H-4’, A), 3.18 (t, *J* = 9.0 Hz, 1H, H-4’, B), 2.40 (s, 3H, C-3-*Me*, B), 2.38 (s, 3H, C-3-*Me*, A).

^13^C NMR (151 MHz, DMSO) δ 182.54 (C-10, A), 182.14 (C-10, B), 164.54 (C-6, B), 160.65 (C-8, B), 158.09 (C-8, A), 146.83 (C-3, A) 136.35 (C-14, B), 135.37 (C-6, A), 134.79 (C-14, A), 124.82 (C-2, B), 122.42 (C-7, A), 121.62 (C-13, A), 120.35 (C-5, A), 118.82 (C-4, B), 118.17 (C-4, A?), 114.68 (C-13, B), 107.30 (C-7, B), 106.31 (C-5, B), 100.68 (C-1’, B), 100.64 (C-1’, A), 77.51 (C-5’, B), 77.34 (C-5’, A), 76.60 (C-3’, B), 76.45 (C-3’, A), 73.24 (C-2’, C-2’, A and B), 69.79 (C-4’, B), 69.54 (C-4’, A), 60.76 (C-6’, B), 60.60 (C-6’, A), 56.09 (C-6-O*Me,* B), 21.48 (C-3-*Me*, A), 21.44 (C-3-*Me*, B).

[α]_D_^22^ = –21.8° (MeOH, c = 0.17).

MS (ESI) *m/z*: [M+Na]^+^ 439.1 and 469.1.

### Cytotoxicity of anthraquinones towards sensitive and drug-resistant cancer cells

As a first step, the cytotoxicity of the main anthraquinones in *R. acetosella* was investigated towards drug-sensitive CCRF-CEM and multidrug-resistant P-glycoprotein-overexpressing CEM/ADR5000 leukemia cells by means of the resazurin assay.

**Figure 2:**
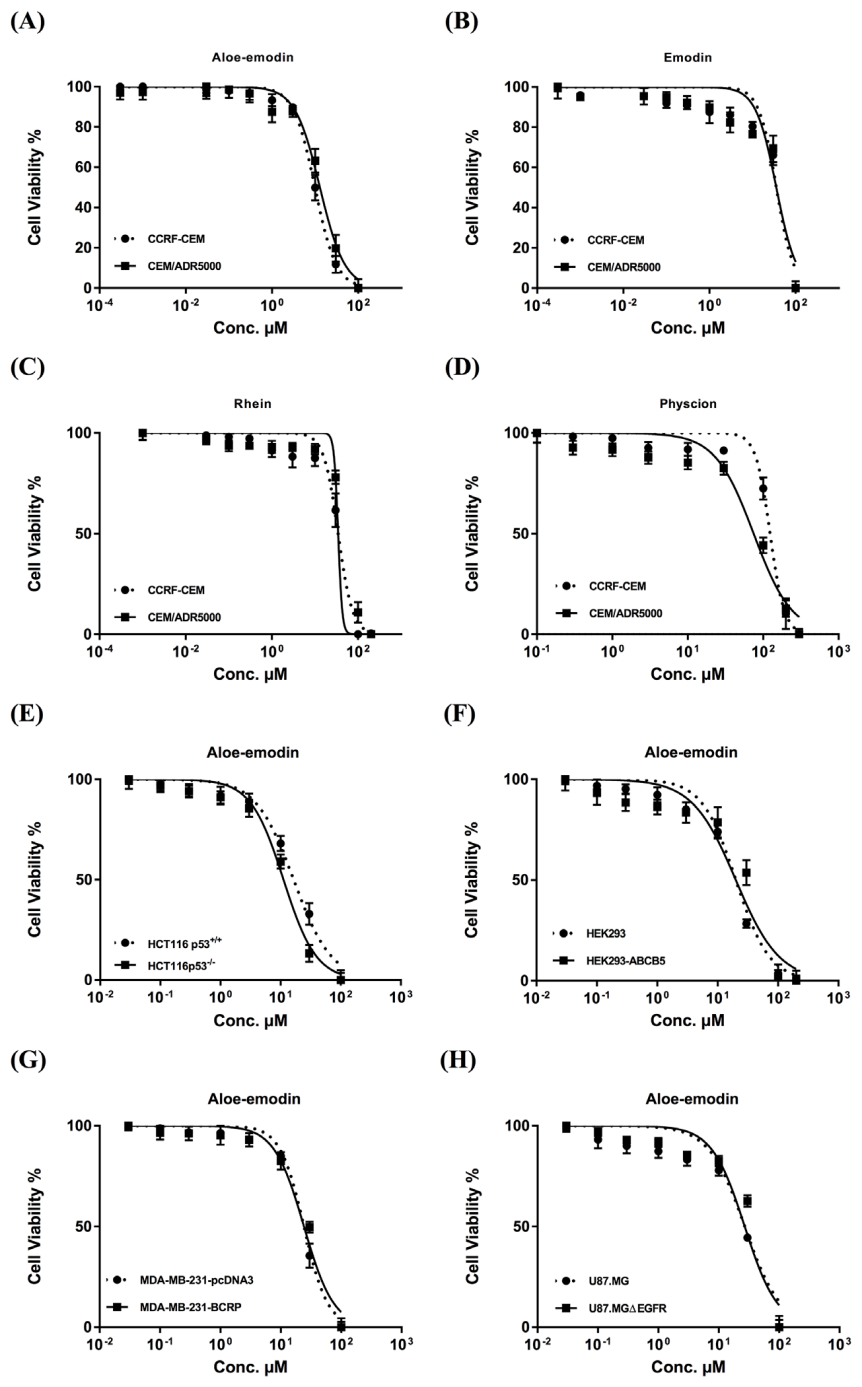
Cytotoxicity of Aloe-emodin **(A)**, emodin **(B)**, rhein **(C)** and physcion **(D)** towards sensitive CCRF-CEM and multidrug-resistant P-glycoprotein-expressing CEM/ADR5000 acute lymphoblastic cells and of Aloe-emodin as the most cytotoxic compound towards HCT116 (*p53*^*+/+*^) colon cancer cells and its knockout clone HCT116 (*p53*^*-/-*^) **(E)**, HEK293 human embryonic kidney cells and its resistant counterpart HEK293/ ABCB5 transfected with a cDNA of *ABCB5* **(F)**, MDA-MB-231-pcDNA3 breast cancer cells and its resistant subline MDA-MB-231-BCRP clone 23 **(G)**, and U87. MG glioblastoma cells and its transfected subline U87.MGΔEGFR, respectively **(H)**. Mean values ± SD of three independent experiments are shown.

**Figure 3:**
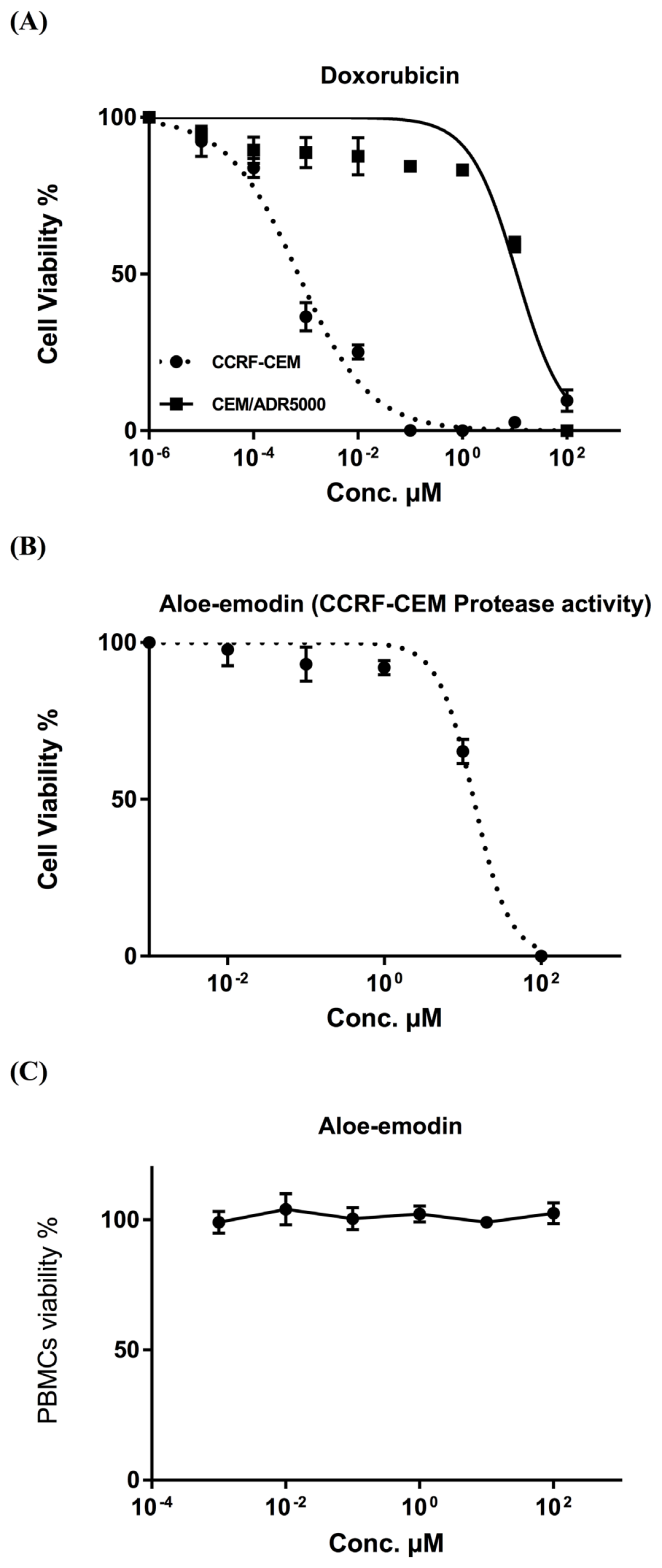
**(A)** Cytotoxicity of doxorubicin towards acute lymphoblastic cells by means for the resazurin assay. Doxorubicin was used as control drug. **(B)** Cytotoxicity of Aloe-emodin towards CCRF-CEM cells by means of protease viability marker assay. This assay was used as independent method for the resazurin assay. **(C)** Toxicity of Aloe-emodin in normal PBMC cells by means of the resazurin assay. These cells served as negative control to prove, whether or not Aloe-emodin inhibits tumor cells in a non-specific manner.

The IC_50_ values of emodin, Aloe-emodin, rhein and physcion were 35.62 μM, 9.872 μM, 34.42 μM and 123.5 μM, respectively, for CCRF-CEM cells and 35.27, 12.85, 46.87 and 74.79 μM, respectively, for CEM/ADR5000 cells (Figure 2). The degrees of resistance were calculated by dividing the IC_50_ values of CEM/ADR5000 cells by the IC_50_ values of CCRF-CEM. Collateral sensitivity (hypersensitivity) of CEM/ADR5000 cells was observed towards physcion (0.61-fold) compared to their corresponding sensitive cells. Aloe-emodin was the most cytotoxic compound among the four anthraquinones with IC_50_ values of 9.872 μM (CCRF-CEM) and 12.85 μM (CEM/ADR5000) (Figure 2). Cytotoxic effects were not measured for compounds 3 and 5, since the isolated amounts were too small to perform for dose response experiments. Doxorubicin as clinically established anticancer drug served as control. The IC_50_ values for doxorubicin were 0.0007 μMand 10.98 μM towards CCRF-CEM and CEM/ADR5000, respectively (Figure 3A). It is worth noting that the IC_50_ value of CEM/ADR5000 cells for Aloe-emodin was similar to that of doxorubicin. As Aloe-emodin exerted the most profound cytotoxicity in sensitive and multidrug-resistant leukemia cell lines, we conducted further experiments solely with Aloe-emodin.

As a next step, we tested the cytotoxicity of Aloe-emodin towards paired cell lines of different tumor types and drug resistance mechanisms. The following cell lines were treated with Aloe-emodin and measured by the resazurin assay:

(1) Breast cancer cells: MDA-MB-231-pcDNA cells and a multidrug-resistant subline transfected with a *BCRP* cDNA (MDA-MB-231-BCRP clone 23).

(2) Embryonic kidney cells: wild type HEK293 cells and a multidrug-resistant subline transfected with an *ABCB5* cDNA (HEK293-ABCB5).

(3) Colon cancer cells: HCT116 cells with wild-type TP53 tumor supressor gene (HCT116 (p53^+/+^)) and HCT116 knockout cells (HCT116 (p53^-/-^)).

(4) Brain tumor cells: wild type U87.MG and a subline transfected with a deletion-activated *EGFR* cDNA (U87.MGΔEGFR).

As shown in Figure 2, the IC_50_ values ranged from 16.47 μM to 22.3 μM for drug-sensitive wild-type cell lines and from 11.19 μM to 33.76 μM for drug-resistant and/or transfected sublines. The IC_50_ values of aloe-emodin towards HCT116(p53^+/+^), U87.MG, MDA-MB-231-pcDNA and HEK293 were 16.47 μM, 21.73 μM, 22.3 μM and 16.9 μM, respectively. For resistant cell lines the IC_50_ values were detected as 11.19 μM (HCT116(p53^-/-^)), 33.76 μM (U87.MGΔEGFR), 26.95 μM (MDA-MB-231-BCRP clone 23) and 25.92 μM (HEK293-ABCB5), respectively. Collateral sensitivity (hypersensitivity) to Aloe-emodin was observed in HCT116 (*p53*^*-/-*^) knockout cells (0.68-fold).

### Cytotoxicity of Aloe-emodin towards CCRF-CEM cells by means of protease viability marker assay

The cytotoxicity of Aloe-emodin was further investigated by means of a protease viability marker assay, in order to rule out any reciprocal interdependence of ROS with the resazurin assay. Since Aloe-emodin induced excessive ROS generation, an interaction with the results of resazurin assay might be suspected. Therefore, we performed a protease viability marker assay, measuring the protease activity within living cells. Aloe-emodin presented strong cytotoxicity towards CCRF-CEM cells with the IC_50_ value of 13.8 μM, which was quite similar to the data gained by the resazurin assay (Figure 3B).

### Toxicity of Aloe-emodin in normal cells

We also investigated Aloe-emodin’s toxicity towards normal cells. Human peripheral mononuclear cells (PMNC) were isolated from fresh blood samples of a healthy donor and tested against various concentrations of Aloe-emodin ranging from 0.001-100 μM.

Interestingly, Aloe-emodin did not show cytotoxic activity towards the normal cells at all concentrations tested (varying from 0.001-100 μM) (Figure 3C). On the contrary, the IC_50_ concentrations needed to kill sensitive and resistance leukemia cell lines as shown in Figure 2 were 9.872 and 12.85 μM, respectively, indicating that the inhibiting effect of aloe-emodin may be tumor-specific.

### Differential transcriptome-wide mRNA expression upon Aloe-emodin treatment

**Figure 4:**
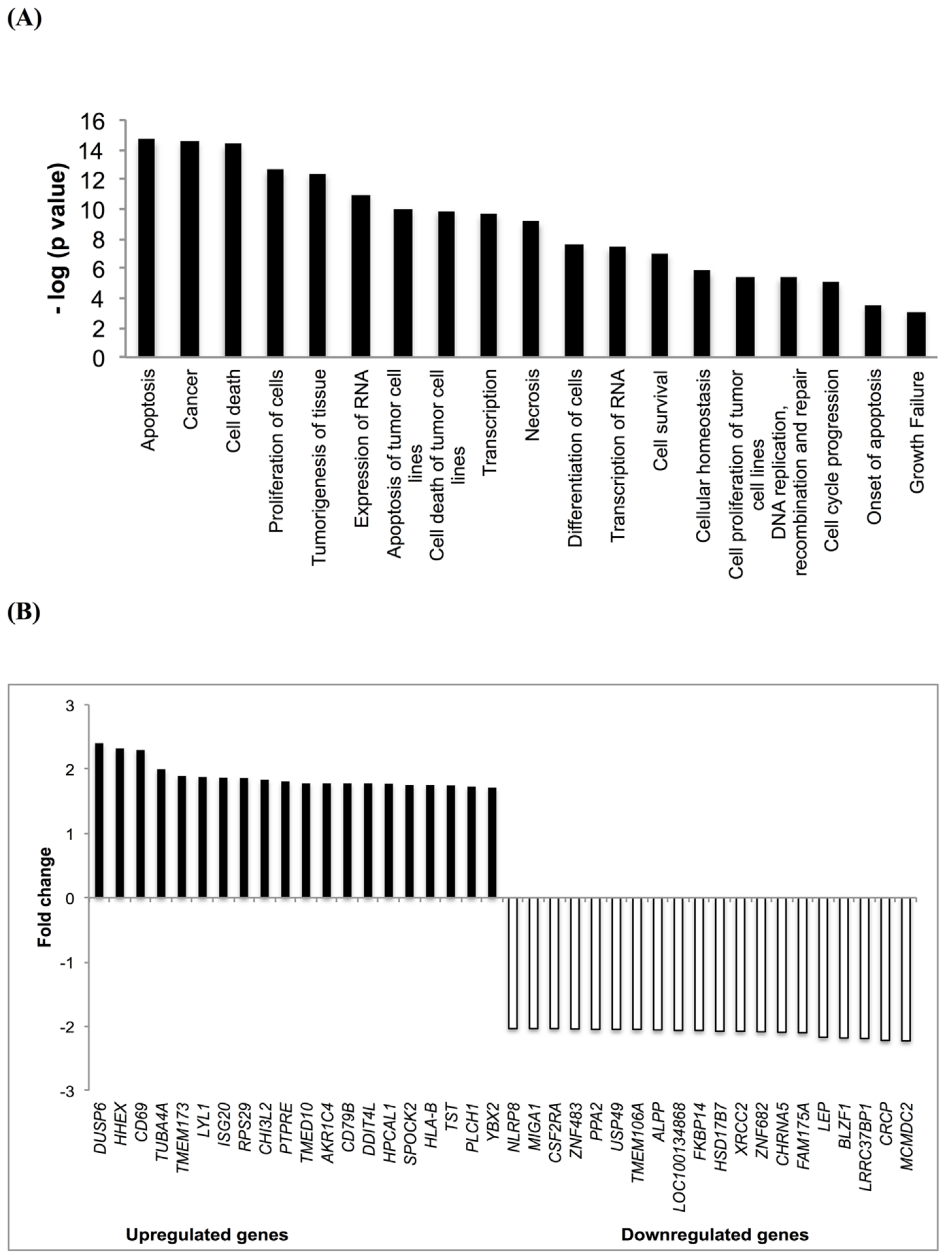
Microarray-based mRNA expression profiling **(A)** Top cellular functions of the most upregulated and downregulated genes of CCRF-CEM cells treated with Aloe-emodin for 48 h identified by Ingenuity Pathway Analysis. **(B)** Top up- and downregulated genes in CCRF-CEM leukemia cells upon treatment with Aloe-emodin for 48 h.

We performed microarray hybridizations to find clues on the possible mechanisms of action of Aloe-emodin. CCRF-CEM cells were treated with the IC_50_ of Aloe-emodin or DMSO for 48 h. Using Chipster software, 1712 genes were deregulated upon Aloe-emodin treatment in comparison with DMSO treatment as control. These genes were subsequently subjected to Ingenuity Pathway Analysis (IPA) to obtain profiles of possibly affected signaling pathways. The most pronounced molecular and cellular functions identified by IPA were: cell death and survival, cellular growth and proliferation, cellular development, gene expression, cellular function and maintenance (Figure 4A).

### Validation of microarray data by qPCR

The most deregulated genes of microarray analyses are shown in Figure 4B. Each two up- or down-regulated genes were selected for qPCR analysis (*DUSP6*, *HHEX*, *MCMDC2*, *CRCP*). Their expression was normalized to *GAPDH*. This gene was selected using the Bestkeeper technique (see Materials and Methods). Then, the fold-change values of Aloe-emodin-treated and untreated samples obtained from microarray hybridization and qPCR were subjected to Pearson correlation test. We obtained a correlation coefficient R-value of 0.989, indicated a high consistency of microarray and qPCR data (Table 1).

**Table 1:** Validation of microarray-based gene expressions by real-time reverse transcription-PCR

Gene name	Microarray data (FC)^*^	qPCR data (FC)
DUSP6	2.403	1.40
HHEX	2.321	2.23
MCMDC2	-2.227	-2.47
CRCP	-2.219	-2.63

R value = 0.989 (Correlation coefficient of mRNA expression values between microarray and qPCR was determined by Pearson correlation test).

^*^ FC: Fold change.

### Detection of reactive oxygen species (ROS)

IPA revealed that the deregulated genes were substantially correlated with apoptosis and other cellular functions including, DNA replication, recombination and repair. Therefore, we assumed that oxidative stress generated by Aloe-emodin may be a reason for DNA damage and ultimately apoptosis.

**Figure 5:**
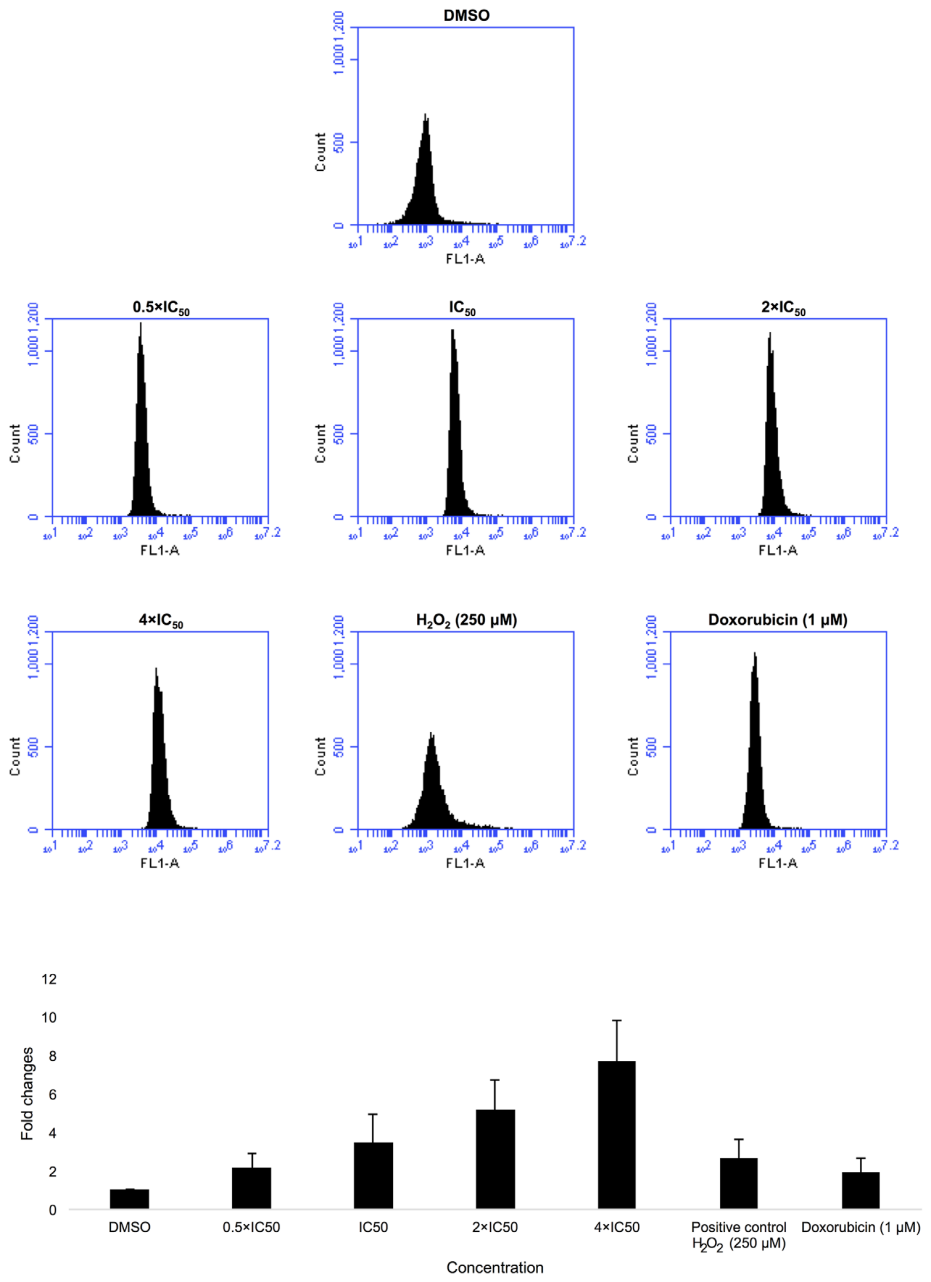
Induction of ROS level in CCRF-CEM cells after treatment with 0,5-, 1-, 2- and 4-fold IC_50_ of Aloe-emodin DMSO has been used as negative control, doxorubicin (1 μM) and H_2_O_2_ (250 μM) as positive controls for 1 h and statistical quantification of ROS level. Mean values ± SD of three independent experiments are shown.

CCRF-CEM cells were treated with 0.5-, 1-, 2- or 4-fold IC_50_ of Aloe-emodin for 1 h. As expected, Aloe-emodin stimulated ROS production in a dose-dependent manner with fold changes ≥ 1.5. Remarkably, Aloe-emodin induced even higher ROS generation than doxorubicin (1 μM) or H_2_O_2_ (250 μM**)** as positive controls (Figure 5).

Excessive ROS production is known to evoke DNA damage in cells [27, 28]. Pathway analysis of microarray results also showed that genes associated with DNA metabolism, replication, recombination and repair were deregulated (Figure 4A). This may imply that Aloe-emodin induces DNA damage. For this reason, we performed alkaline comet assay to detect single and double-stranded DNA damage.

### Comet assay

**Figure 6:**
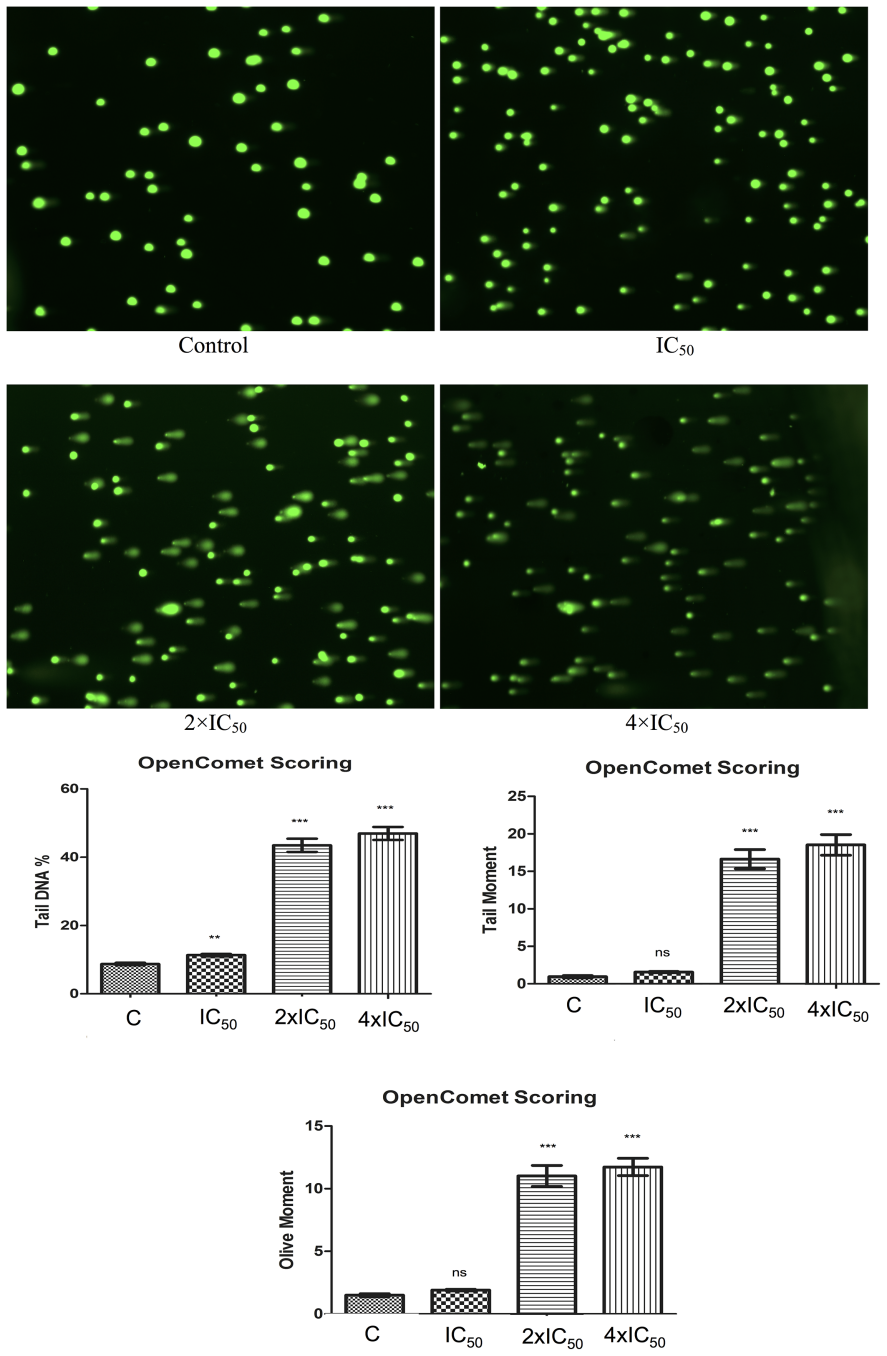
Induction of DNA damage by Aloe-emodin in CCRF-CEM cells Cells were incubated with different concentration of Aloe-emodin for 24 h. DNA damage was measured by the Comet assay. Representative pictures were shown above. Three parameters were detected including tail DNA %, tail moment and olive moment. Tail and olive tail movement were presented in arbitrary units. Results were presented as mean ± SEM of at least 50 cells for each. *ns* not significant, ^**^p < 0.005; ^***^p < 0.0001 as compared to control cells. ^*^P < 0.05 compared with DMSO. Mean values ± SEM of three independent experiments are shown.

Pathway analysis showed that DNA metabolism comprising DNA replication, recombination and repair was deregulated. Therefore, we tested Aloe-emodin’s effect on the DNA by treating CCRF-CEM cells with 1-, 2- or 4-fold IC_50_ of Aloe-emodinfor 24 h. DMSO-treated cells served as negative control. Aloe-emodin indeed induced comet tails and increased percentages of tail DNA. We additionally monitored DNA migration with tail and olive tail movements and concluded that Aloe-emodin induced DNA damage in a dose-dependent manner (Figure 6).

### Cell cycle analysis

**Figure 7:**
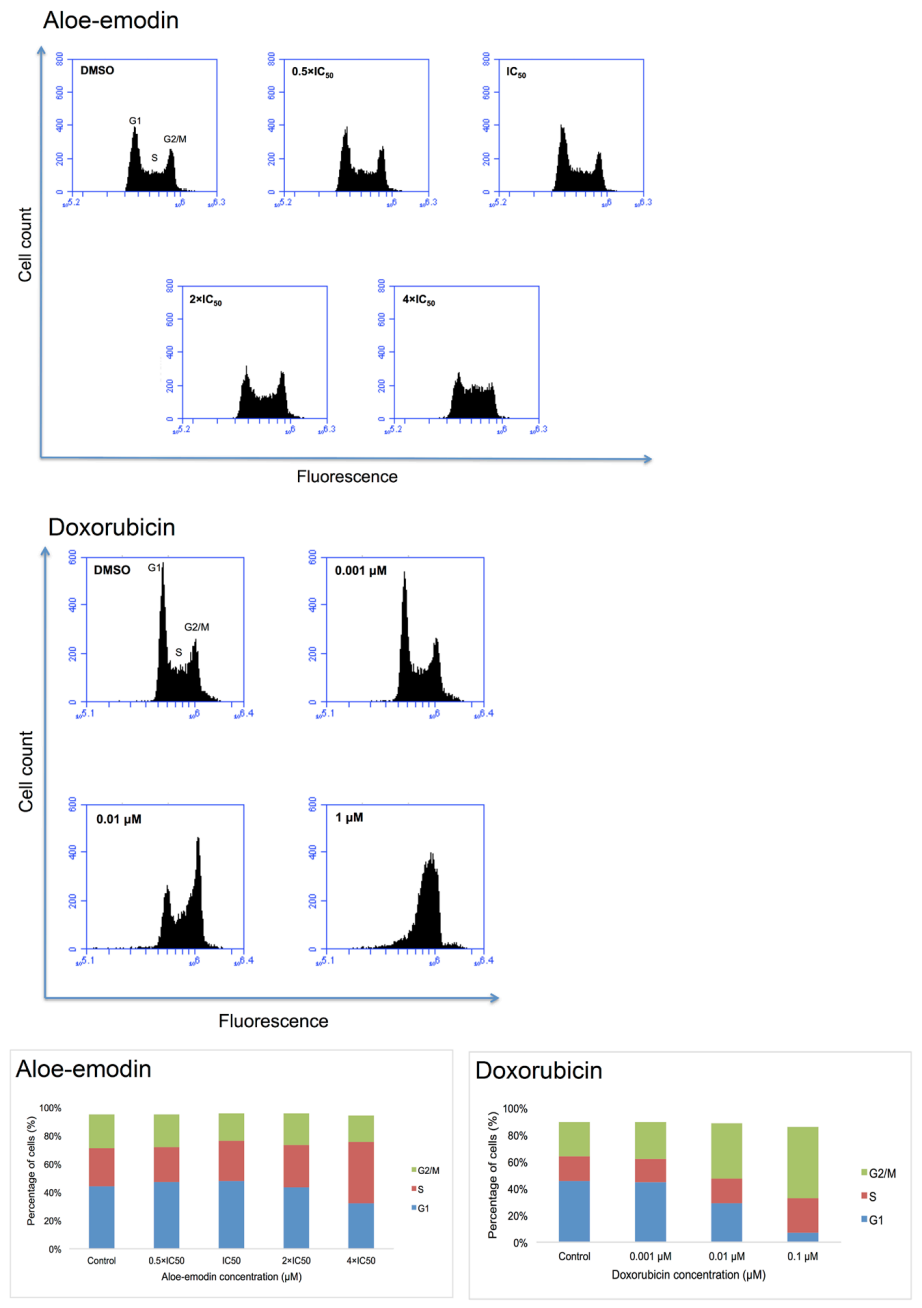
DNA histograms and cell cycle distribution of CCRF-CEM cells treated with indicated concentrations of Aloe-emodin and doxorubicin, respectively for 24 h

Pathway analyses of microarray data indicated that genes associated with cell cycle progression were deregulated by Aloe-emodin. Therefore, we investigated whether Aloe-emodin may cause cell cycle arrest. We treated CCRF-CEM cells with 0.5-, 1-, 2- and 4-fold IC_50_ of Aloe-emodin for 6, 24, 48 and 72 h, respectively. Indeed, the cells showed a clear arrest in the S-phase of the cell cycle upon treatment for 24 h with the 4-fold IC_50_ of Aloe-emodin (Figure 7). Doxorubicin was used as positive control. Treatment with 0.01 μM and 0.1 μM doxorubicin for 24 h induced both S- and G2/M-arrest (Figure 7). The S-phase arrest induced by Aloe-emodin may be a consequence of oxidative stress and DNA damage.

### Measurement of mitochondrial membrane potential

DNA damage causes apoptosis [28]. Numerous apoptosis-regulating genes were deregulated in the microarray experiments, which represents another clue for apoptosis induction following Aloe-emodin exposure. An early event in mitochondria-driven apoptosis is the breakdown of the mitochondrial membrane potential (MMP) [29, 30]. Mitochondria control cell death by releasing cytochrome *c* to the cytosol and followed by activating caspases [31].

**Figure 8:**
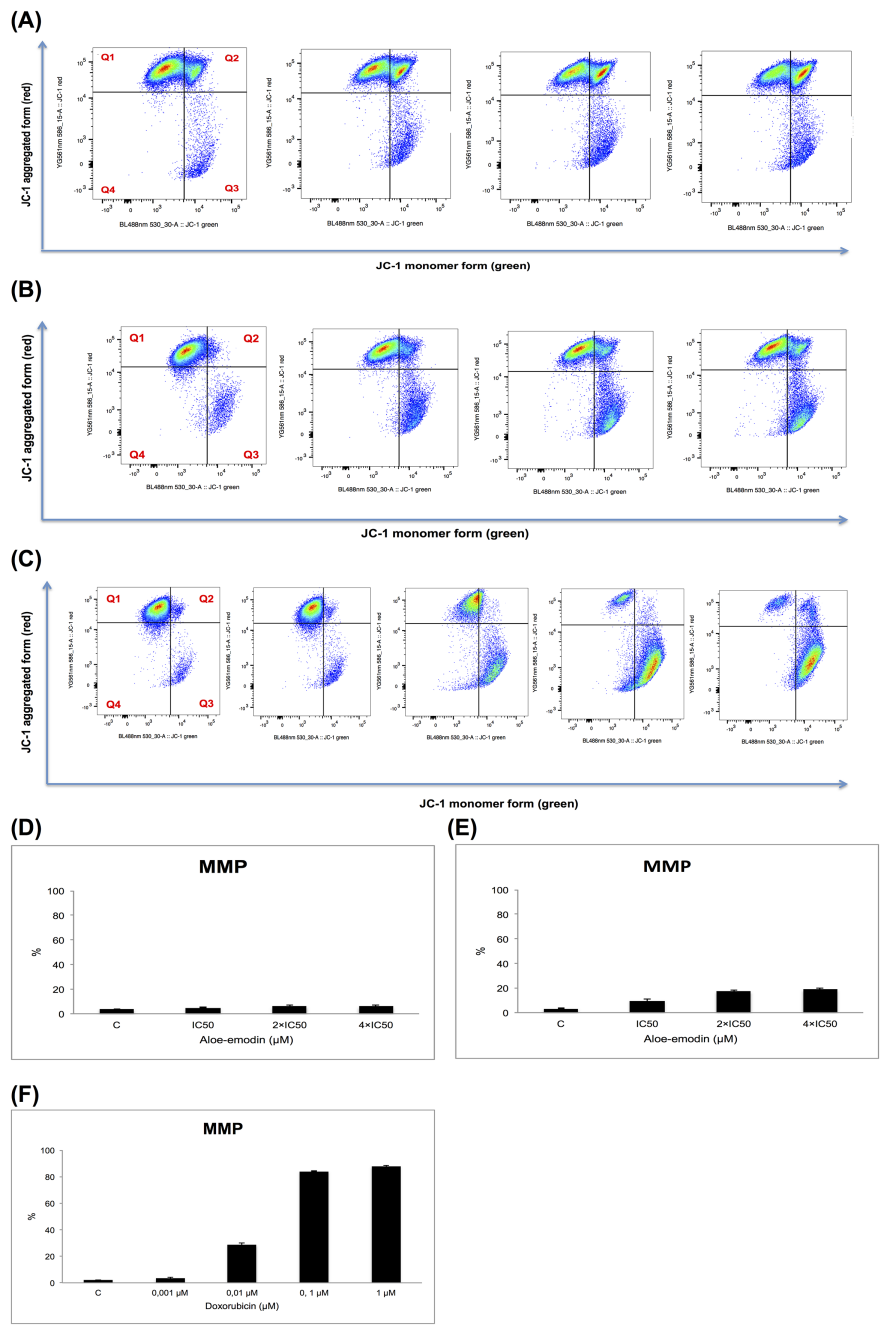
Disruption of the mitochondrial membrane potential by Aloe-emodin and doxorubicin, respectively in CCRF-CEM cells Cells were treated with or without DMSO as negative control and 1-, 2- and 4-fold IC_50_ of Aloe-emodin, respectively for 24 h **(A)** or for 48 h **(B)** or with 0.001, 0.01, 0.1 and 1 μM doxorubicin for 48 h as positive control **(C)** and stained by JC-1. Intact cells mainly displayed the J-aggregated form with red fluorescence (Q1) and cells with loss of MMP showed JC-1 monomers with green fluorescence (Q3). Statistical results of the cell population in Q3, which was defined as disruption of mitochondrial membrane potential with Aloe-emodin treatment for 24 h **(D)**, 48 h **(E)** or doxorubicin treatment for 48 h **(F)**, respectively. Mean values ± SD of three independent experiments are shown.

We analyzed MMP alterations in CCRF-CEM cells. Therefore, we treated the cells with 1-, 2- and 4-fold IC_50_ of Aloe-emodin, respectively, and incubated for 24 and 48 h. Doxorubicin (0.001, 0.01, 0.1 and 1 μM) was tested as positive control for 48 h. CCRF-CEM cells stained with the MMP-specific dye JC-1 revealed a shift from red to green fluorescence following treatment with 2- and 4-fold IC_50_ of Aloe-emodin (Figure 8A, 8B, 8D and 8E) and 0.01, 0.1, and 1 μM doxorubicin for 48 h (Figure 8C and 8F) indicating MMP depolarization.

### Detection of apoptosis and necrosis

**Figure 9:**
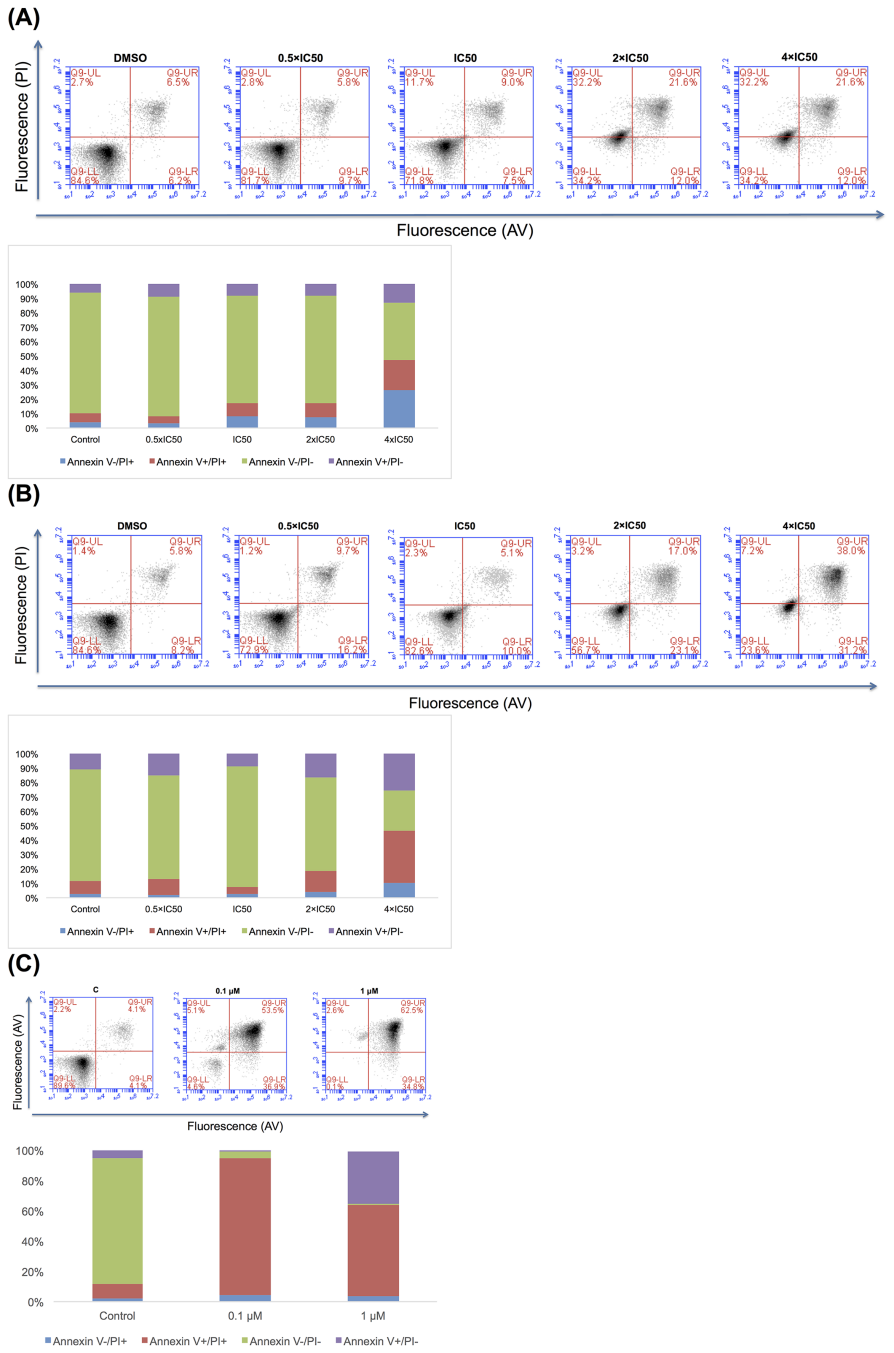
Apoptosis effect in CCRF-CEM cells of Aloe-emodin for 72 h **(A)** and 96 h **(B)** and of doxorubicin for 72 h **(C)**

We performed annexin V/PI staining to detect apoptotic or necrotic cell death in CCRF-CEM cells. The gated cells showed different populations corresponding to viable and non-apoptotic (annexin V–PI–), early (annexin V+PI–), and late apoptotic as well as early (annexin V+PI+) and late necrotic (annexin V–PI+) cells. Doxorubicin inclined late apoptosis and early necrosis after treatment with 0.1 μM or late necrosis after treatment with 1 μM for 72 h (Figure 9). Aloe-emodin induced early and late apoptosis as well as early and late necrosis after treatment with the 4-fold IC_50_ for 72 h or early and late apoptosis as well as early and late necrosis after treatment with 2-or 4-fold IC_50_ for 96 h (Figure 9).

### COMPARE and hierarchical cluster analyses of transcriptome-wide mRNA expression in untreated cell lines

The mRNA microarray data of the NCI tumor cell line panel have been deposited at the NCI website (http://dtp.cancer.gov/databases_tools/default.htm). We applied COMPARE analyses to generate rank-ordered lists of genes expressed in the NCI cell lines. As previously described [32, 33], every gene of the NCI microarray database was ranked for similarity of its mRNA expression to the log_10_IC_50_ values for the compound under investigation (*i.e.,* Aloe-emodin). A scale index of correlation coefficients (*R*-values) was created to derive COMPARE rankings.

**Figure 10:**
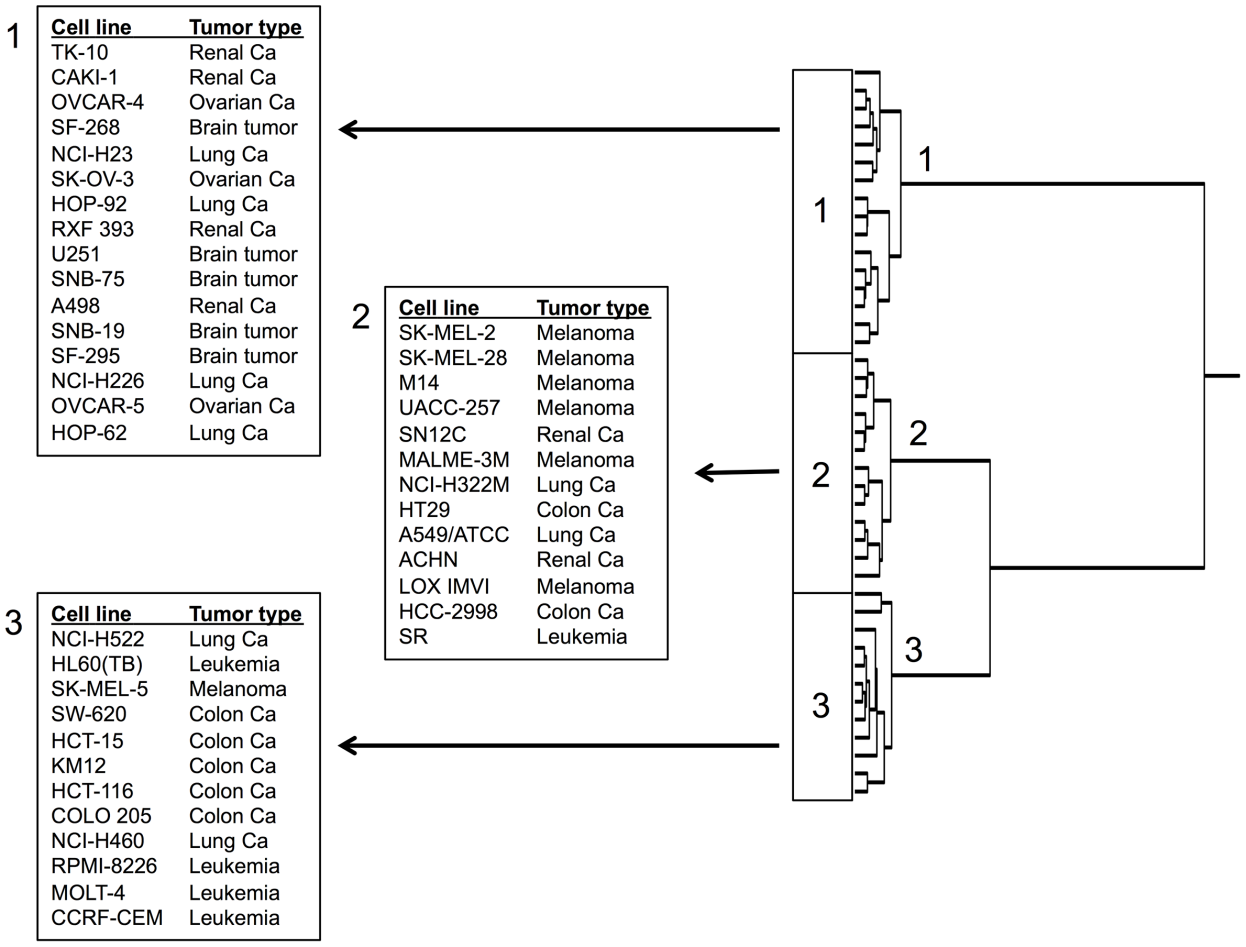
Hierarchical cluster analysis of microarray-based mRNA expression of genes for Aloe-emodin The dendrograms show the clustering of the NCI cell line panel according to the degrees of relatedness between cell lines.

The mRNA expression data of the cell lines were subjected to hierarchical cluster analysis. Each three main cluster branches appeared in the dendrogram for Aloe-emodin (Figure 10). We examined the distribution of sensitive or resistant cells to Aloe-emodin by using the χ^2^ test.

To perform the χ^2^-test, we defined the cell lines as being sensitive or resistant to Aloe-emodin. The log_10_IC_50_ value of Aloe-emodin for all cell lines (−4.35 M) served as cut-off threshold. Cell lines with log_10_IC_50_ values below this threshold were defined as sensitive, cell lines above this threshold as resistant. Cluster 1 and cluster 3 contained mainly sensitive and resistant cell lines, respectively, whereas cluster 2 was of a mixed type. This distribution of sensitive and resistant cell lines was statistically significant (χ^2^ test*, P* = 5.90×10^-7^; Table 2). This implies that the expression of this set of genes caused dendrogram branching in a way that gene expression predicted the inherent cellular responsiveness to Aloe-emodin in a statistically significant manner.

**Table 2:** Separation of clusters of NCI cell line panel obtained by hierarchical cluster analysis shown in Figure 10 in comparison to drug sensitivity^a^

	Partition	Cluster 1	Cluster 2	Cluster 3
Sensitive	< -4.35 M	0	8	12
Resistant	> -4.35 M	16	5	0
chi-square test: p= 5.90 ×10^-7^				

^a^The median log_10_IC_50_ value (−4.35 M) for each compound was used as a cutoff to separate tumor cell lines as being “sensitive” or “resistant”.

The analysis of microarray data showed that genes involved in signal transduction, apoptosis, nucleic acid metabolism etc. seemed to have a role for inherent resistance responsiveness of tumor cells towards Aloe-emodin (Table 3).

**Table 3:** Correlation of constitutive mRNA expression of genes identified by COMPARE analyses with the log_10_IC_50_ values of Aloe-emodin for the NCI tumor cell lines

COMPARE coefficient	Experimental ID	GeneBank accession	Gene symbol	Name	Function
0.641	29457	Y00978	*DLAT*	Dihydrolipoamide S-acetyltransferase	Transferase activity; links the glycolytic pathway to the tricarboxylic cycle
0.614	23142	D50929	*EIF3A*	Eukaryotic translation initiation factor 3, subunit A	Involved in apoptosis of synovial fibroblasts
0.613	31751	AF047042	*CS*	Citrate synthase	Poly(A) RNA binding and citrate (Si)-synthase activity; mitochondrial targeting
0.584	25141	D21851	*LARS2*	Leucyl-tRNA synthetase 2, mitochondrial	Nucleotide binding and aminoacyl-tRNA editing activity
0.581	31188	AF081280	*NPM3*	Nucleophosmin/nucleoplasmin 3	Poly(A) RNA binding
0.57	24945	S79522	*RPS27A*	Ribosomal protein S27a	Poly(A) RNA binding and structural constituent of ribosome
0.561	22197	AB011136	*KIAA0564*	KIAA0564	ATPase activity
0.558	25753	AB018307	*SUPT7L*	Suppressor of Ty 7 (*S. cerevisiae*)-like	Transcription coactivator activity and histone acetyltransferase; role in chromatin activation, transcriptional regulation, and DNA damage repair
0.551	31083	D50925	*PASK*	PAS domain containing serine/threonine kinase	Transferase activity; protein tyrosine kinase activity
0.545	28659	D26488	*WDR43*	WD repeat domain 43	Poly(A) RNA binding and binding
0.533	22297	X79563	*RPS21*	Ribosomal protein S21	Poly(A) RNA binding and protein N-terminus binding
0.524	30065	U94703	*POLG2*	Polymerase (DNA directed), γ2, accessory subunit	Identical protein binding and DNA-directed DNA polymerase activity
0.523	23131	AI541050	*NDUFB8*	NADH dehydrogenase (ubiquinone) 1 β subcomplex, 8, 19 kDa	NADH dehydrogenase (ubiquinone) activityand NADH dehydrogenase activity
0.521	30499	X16396	*MTHFD2*	Methylenetetrahydrofolate dehydrogenase (NADP^+^ dependent) 2, methenyltetrahydrofolate cyclohydrolase	Magnesium ion binding and formate-tetrahydrofolate ligase activity
0.516	29266	D79989	*AGAP2*	ArfGAP with GTPase domain, ankyrin repeat and PH domain 2	GTP binding and GTPase activator activity mediates anti-apoptotic effects of nerve growth factor is overexpressed in cancer cells, and promotes cancer cell invasion
0.515	32195	M92439	*LRPPRC*	Leucine-rich PPR-motif containing	Poly(A) RNA binding and ubiquitin protein ligase binding
0.514	25646	AF067139	*NDUFS3*	NADH dehydrogenase (ubiquinone) Fe-S protein 3, 30kDa (NADH-coenzyme Q reductase)	Poly(A) RNA binding and ubiquitin protein ligase binding; transcriptional regulation of both nuclear and mitochondrial genes
0.506	25108	D80007	*PDCD11*	Programmed cell death 11	Nucleic acid binding and transcription factor binding; required for rRNA maturation and generation of 18S rRNA
0.506	31640	L49380	*SF1*	Splicing factor 1	Nucleic acid binding and RNA binding; plays a role in nuclear pre-mRNA retention and transcriptional repression
0.502	32348	U66615	*SMARCC1*	SWI/SNF related, matrix associated, actin dependent regulator of chromatin, subfamily c, member 1	Chromatin binding and RNA polymerase II core promoter proximal region sequence-specific DNA binding
-0.606	27140	AL096739	*GALNT10*	UDP-N-acetyl-α-D-galactosamine:polypeptide N-acetylgalactosaminyltransferase 10 (GalNAc-T10)	Carbohydrate binding and polypeptide N-acetylgalactosaminyltransferase activity
-0.581	24659	AB023175	*POFUT2*	Protein O-fucosyltransferase 2	Fucosyltransferase activity and peptide-O-fucosyltransferase activity
-0.547	28663	AB020689	*TBC1D9*	TBC1 domain family, member 9 (with GRAM domain)	Calcium ion binding and GTPase activator activity
-0.546	24243	AI547262	*ATP6V0E1*	ATPase, H^+^ transporting, lysosomal 9 kDa, V0 subunit e1	Transporter activity and proton-transporting ATPase activity, rotational mechanism; encodes a component of vacuolar ATPase (V-ATPase) which is necessary for diverse intracellular processes
-0.545	32200	Z47087	*SKP1*	S-phase kinase-associated protein 1	Ubiquitin-protein transferase activity; encodes an essential component of SCF complex which mediates ubiquitination of proteins involved in cell cycle progression, signal transduction and transcription
-0.542	31737	AA477898	*ITM2B*	Integral membrane protein 2B	β-amyloid binding plays a regulatory role in processing of β-amyloid A4 precursor protein (APP); inhibitor of β-amyloid peptide aggregation and fibrils deposition
-0.538	23424	L07738	*CACNG1*	Calcium channel, voltage-dependent, γ subunit 1	Voltage-gated calcium channel activity; role in excitation-contraction coupling
-0.536	29496	M88458	*KDELR2*	KDEL (Lys-Asp-Glu-Leu) endoplasmic reticulum protein retention receptor 2	ER (endoplasmic reticulum) retention sequence binding and KDEL (Endoplasmic Reticulum Protein Retention Receptor 2) sequence binding.
-0.532	22897	AF063002	*FHL1*	Four and a half LIM domains 1	Ion channel binding
-0.531	24272	AB007144	*DAPK3*	Death-associated protein kinase 3	Protein homodimerization activity and transferase activity; role in induction of apoptosis
-0.531	30005	AB020640	*CAMTA1*	Calmodulin binding transcription activator 1	Transcriptional activator; may act as tumor suppressor
-0.527	29746	D86983	*PXDN*	Peroxidasin homologue (*Drosophila*)	Heme binding and peroxidase activity; involved in extracellular matrix formation; may function in the physiological and pathological fibrogenic response in fibrotic kidney
-0.526	29587	AL049957	*CD59*	CD59 molecule, complement regulatory protein	Complement binding; encodes a cell surface glycoprotein that regulates complement-mediated cell lysis; involved in lymphocyte signal transduction
-0.524	22562	M83088	*PGM1*	Phosphoglucomutase 1	Magnesium ion binding and phosphoglucomutase activity; participates in both breakdown and synthesis of glucose
-0.523	20761	L25081	*RHOC*	Ras homolog gene family, member C	GTP binding and signal transducer activity; overexpression is associated with tumor cell proliferation and metastasis
-0.522	30218	D63475	*AP2M1*	Adaptor-related protein complex 2, μ 1 subunit	Transporter activity and low-density lipoprotein particle receptor binding
-0.522	26170	H93123	*VAMP3*	Vesicle-associated membrane protein 3 (cellubrevin)	SNARE binding and syntaxin-1 binding
-0.516	20862	L77886	*PTPRK*	Protein tyrosine phosphatase, receptor type, K	Protein kinase binding and protein tyrosine phosphatase activity; regulation of processes involving cell contact and adhesion (growth control, invasion, and metastasis)
-0.511	32199	AL096879	*TMEM184B*	Transmembrane protein 184B	May activate the MAP kinase signaling pathway
-0.51	27189	Z24727	*TPM1*	Tropomyosin 1 (α)	Actin binding and cytoskeletal protein binding; suppresses anchorage-independent growth

Positive correlation coefficients indicate direct correlations to log_10_IC_50_ values, negative ones indicate inverse correlations. Information on gene functions was taken from the OMIM database (NCBI, Bethesda, MD, USA) [78] and from the GeneCards database of the Weizman Institute of Science (Rehovot, Israel) [79].

## DISCUSSION

In the present investigation, we analyzed the modes of action and determinants of resistance of cancer cells to chemical constituents of *R. acetosella* with special emphasis to Aloe-emodin. The isolation of phytochemicals from medicinal plants represents a state-of-the-art procedure in pharmacognosy. *R. acetosella* contains anthraquinones, flavonoids and other phenolic compounds. We decided to focus on anthraquinones because of their striking chemical similarity to anthracyclines as clinically well-established anticancer drugs since decades. Because of this chemical relationship, we suggested cytotoxic acivity against cancer cells.

Additionally, the cytotoxic activity of anthaquinones provides a rationale explanation that *Rumex* species have been traditionally used to treat cancer [21, 22, 34]. A major anthraquinone aglycone of *Rumex* and other genera (*e.g. Aloe, Rheum* and *Rhamnus*) is Aloe-emodin [35, 36]. A main motivation to investigate the molecular mechanisms of Aloe-emodin was its activity against drug-resistant tumor cells. There are hundreds, if not thousands of cytotoxic phytochemicals from medicinal plants, which never reached clinical application. What is really needed to our opinion, are novel substances that are better than the established anticancer drugs. Aloe-emodin is not only outstanding to suppress tumor growth *in vivo* as previously demonstrated [37–39], but this compound was also able to kill tumor cells, which are resistant to standard anticancer agents as shown here. The development of resistance to anticancer drugs poses a major obstacle to successful chemotherapy, and innumerous patients died because of their refractory and resistant tumors. For this reason, there is a high demand for novel compounds with activity against otherwise drug-resistant tumors. Aloe-emodin may have the potential as new drug, because of its property to kill otherwise refractory.

We focused on resistance phenotypes, which are characterized by broad cross-resistance not only towards single, but also towards many chemically and functionally different cytostatic drugs. These multiple drug resistance (MDR) phenomena are frequently mediated by drug efflux pumps of the ATP-binding cassette (ABC) type as well as activated oncogenes or inactivated tumor suppressor genes. We have chosen three ABC-transporters, *i.e.,* P-glycoprotein (MDR1/ABCB1) and breast cancer resistance protein (BCRP/ABCG2) as well-known MDR-mechanisms and ABCB5 as novel efflux transporter relevant for cancer stem-like cells. Oncogenes and tumor suppressor genes do not only contribute to carcinogenesis, but also confer drug resistance [40]. We selected the epidermal growth factor receptor (EGFR) and the tumor suppressor gene p53 for our investigations.

After isolation of several anthraquinones, we concentrated on Aloe-emodin as the most cytotoxic compound in our investigation. Interestingly, HCT116 (p53^-/-^) cells even exhibited collateral sensitivity to Aloe-emodin. In P-glycoprotein-overexpressing cells, collateral sensitivty has been explained by futile cycles of ATP cleavage by the efflux transporter and transient depletion of cellular ATP stores ultimately leading to cell death [41]. However, the underlying mechanisms of collateral sensitivity in p53-knockout cells is completely unknown as yet and deserves further investigation.

The analysis of resistant cell lines provided us first clues on the mechanisms of action of Aloe-emodin. However, cell lines of different tumor types may respond differently to Aloe-emodin according to their individual gene expression profiles. Therefore, we wanted to find out, which tumor types reacted better or worse to Aloe-emodin. To address this question, we performed COMPARE and hierarchical cluster analysis of transcriptome-wide microarray-based mRNA hybridizations in a panel of 60 tumor cell lines of DTP (NCI, USA) (http: dtp.cancer. gov). DTP assessed to more than 88.000 pure compounds and more than 34.000 crude extracts against the panel of 60 human tumor cell lines as of yet. Interestingly, the pattern of cellular sensitivity and resistance of these cell lines to established anticancer drugs as well as investigational cytotoxic compounds correlated with their molecular target expression [42, 43]. This approach has been frequently applied by us and others in recent years [44–48]. Here, we used this approach to identify genes, whose expression correlated with sensitivity or resistance of the cell lines with Aloe-emodin.

An intriguing result of hierarchical cluster analyses was that a set of only 40 genes out of the entire transcriptome was sufficient to determine, whether a cell line was sensitive or rather resistant to Aloe-emodin. This is a remarkable result, because the IC_50_ values of the cell lines had not been prior included into the calculations for the cluster analyses. The implication of this result is that sensitivity or resistance of a drug in tumor cells can be predicted based on the gene expression profile alone. It can be speculated that such an approach could be translated to the clinical setting for the individualized treatment of cancer patients.

The following scenario can be envisaged: If a tumor is resistant to most standard drugs, gene expression profiling could help to predict, which cytotoxic phytochemical is still active in this tumor. Before clinical treatment with Aloe-emodin can be realistically considered for routine treatment, robust gene expression profiles of patients’ biopsies have to be determined that reliably predict tumor sensitivity towards Aloe-emodin. The present paper may open avenues for cancer precision medicine with Aloe-emodin once this compound could be clinically established. More analyses are necessary before this concept can be realized in clinical everyday practice. Nevertheless, our results represent a proof-of-principle that natural products in general represent a promising resouce for novel anicancer drugs, which might be implemented in future treatment strategies.

Two conditions are necessary to realize this concept of individualized therapy with natural procuts from medicinal plants (1) the panel of available cytotoxic phytochemicals should be large enough to choose the right compound for the right patient; (2) therapeutically relevant genes have to be separated from non-relevant background noise in the gene expression profile. It is a frequently made observation that many genes that are deregulated upon drug treatment do not bear functional relevance for the corresponding drug, but are just concomitant non-causative alterations. Recently commercial low density arrays have been marketed that carry only genes with causative relevance for the response of tumors to standard chemotherapy. Although gene expression profiles have been generated in cell lines for many phytochemicals [49, 50], clinical validation has not been done yet. This represent an important prerequisite to establish natural product-based cancer therapy in the future.

For Aloe-emodin, the range of genes with different functions is remarkably diverse. Genes operating in signal transduction, apoptosis, nucleic acid metabolism and other pathways were identified by COMPARE analysis. This gene expression profile resembles the molecular architecture of cell lines that have not been pretreated with Aloe-emodin. This experimental setting is characteristic for a phenomenon clinically known as inherent or primary resistance. While some tumors will respond to chemotherapy, others are non-responsive, although they have never been pretreated with anticancer drugs.

In addition to primary resistance, an initially sensitive tumor can acquire resistance upon repeated chemotherapy cycles, which was termed acquired or secondary resistance. Under laboratory conditions, it is possible to compare gene expression profiles of treated and non-treated cells. The resulting differentially expressed genes can be used to generate testable hypothesis on the molecular modes of action and determinants of resistance of cytotoxic compounds. There are numerous examples in the literature for the validity of this concept [51–54].

The results as well as the genes obtained by COMPARE and cluster analyses indicate that Aloe-emodin acted by multiple mechanisms against cancer cells. Multi-specificity represents a typical feature of many - if not all - natural products [55]. Based on the microarray data of differentially expressed genes between treated and non-treated cells, we assumed that Aloe-emodin generates ROS, which leads to DNA damage and cell cycle arrest. As a consequence, the mitochondrial pathway of apoptosis is induced as shown by disruption of the mitochondrial membrane potential and annexin V/PI staining.

Findings of other authors confirm our point of view: Aloe-emodin induced DNA-damage *in vitro* [56–58]. In animal experiments, it inclined DNA damage after two oral application [38]. These data fit to the observed cell cycle disturbations (i.e., S- and G2/M-arrest) as well as apoptosis upon Aloe-emodin exposure [59].

In addition to the elucidation of mechanisms of action and determinants of resistance to Aloe-emodin, our molecular pharmacological data substantiate the therapeutic application of *R. acetosella* against tumors in traditional medicine.

Nevertheless, it should be pointed out that the traditional use of *R. acetosella* as anticancer remedy cannot be explained by Aloe-emodin alone and that presumably other phytochemicals contribute to the bioactivity of this plant. Several modes of action can be envisioned:

(1) One substance is the main active compound and other compounds in the plant support its actions, *e.g*. as solvent mediators.

(2) Several compounds reveal bioactivity against one or several therapeutic targets. The substances mutually supplement each other in additive or synergistic manner.

(3) The main compound reveals not only activity against diseased cells and tissues but also against normal tissues leading to side effects. Concomitant compounds in the plant dampen the side effects.

Based on the results of the present investigation, further analyses are warranted to clarify, which of these possibilities are realized in*R. acetosella*. This final goal is to understand the full bioactivity of this plant to utilize its full potential for rationale phytotherapy of cancer.

## MATERIALS AND METHODS

### Plant material

*R. acetosella* was collected from Camlidere (Ankara, Turkey) in May 2012. A voucher specimen has been deposited in the Herbarium of Faculty of Pharmacy, Hacettepe University, Ankara, Turkey (HUEF: 13005).

### General phytochemical procedure

Normal phase column chromatography and reverse phase column chromatography were run on silica gel 60 (0.063-0.200 mm, Merck, Darmstadt, Germany) and RP-18 silica gel (40-63 μM, Merck, Darmstadt, Germany), respectively. Sephadex LH-20 (Sigma, Sweden) was used for separation of the compounds based on their molecular size. Thin layer chromatography (TLC) was applied on silica gel 60 F_254_ precoated plates (Merck, Darmstadt, Germany). The spots were observed using an UV lamp at 254 and 365 nm, followed by spraying with 1% vanillin/H_2_SO_4_ and then heating at 110°C.

Nuclear magnetic resonance (NMR) spectra were recorded on an Avance III 600 (Bruker) using 5 mm probe heads at a temperature of 23°C. The ^1^H and ^13^C chemical shifts were referenced to the residual/deuterated solvent (*e.g.*, for MeOD, δ = 3.31 and 49.00 ppm for 1H and 13C NMR, respectively) and reported in parts per million (ppm, δ) relative to tetramethylsilane (TMS, δ = 0.00 ppm). Coupling constants (*J*) are reported in Hz, and the splitting abbreviations used were: s, singlet; d, doublet; t, triplet; m, multiplet; br, broad; and combinations thereof. High-resolution masses (ESI) were recorded on a Q-ToF-Ultima 3 instrument (Waters) with LockSpray™ interface and a suitable external calibrant. Optical rotations were measured with a Perkin–Elmer 241 polarimeter at 589 nm. Infrared spectra were recorded as FT-IR spectra on a Tensor 27 spectrometer (Bruker) using a diamond ATR unit and are reported in terms of frequency of absorption (ν, cm^−1^).

### Extraction and isolation of natural substances

Roots of *R. acetosella* (529.68 g) were dried on air, grinded and extracted with methanol (10 L × 7) at 40°C under reflux for 72 h. The extract was filtered and evaporated under vacuum and 83.26 g crude extract was gained. We used normal phase silica gel column chromatography (SK), reverse phase column chromatography including vacuum liquid chromatography (VLC) or medium pressure liquid chromatography (MPLC) and Sephadex column chromatography with isocratic CH_3_OH elution (SPH) to isolate pure compounds.

Initially, the crude extract was processed by SK-1 gradient elution from CH_2_Cl_2_: CH_3_OH (95:5) to CH_2_Cl_2_:CH_3_OH:H_2_O (60:40:4). Fraction (27-28) of SK-1 was further processed by SK-16 accompanied by CH_3_COOC_2_H_5_:CH_3_OH:H_2_O gradient elution from (100:5:2) to (100:17:13). Fraction (10-14) of SK-16 was subjected to SPH-15. The obtained fraction (13-16) gained was re-conducted on SPH-16 to get fraction (15-26), which was enriched with compound 1. This fraction was subsequently applied to VLC consecutively to get fraction VLC-9/(16-17) as pure compound (**Compound 1**, 21.3 mg).

Similar purification steps as for compound 1were performed, until we obtained fractions of SPH-15. SPH-15/(5-7) was applied to SPH-19. Fraction (7-11) from SPH-19 was subjected to VLC to obtain VLC-10/(15-16) as pure compound (**Compound 2**, 10 mg).

Fraction 15-20 from SK-16 was run on successive SPH columns to get the fraction (8-13) from SPH-21. Subsequently, the fraction was processed by VLC-11 with CH_3_OH:H_2_O (20:80) isocratic elution. Then, the fraction (20-24) from VLC being virtually pure was finally applied to preparative TLC to yield completely pure compound (**Compound 3**, 12.4 mg).

The fraction SK-1/(35-36) as one of the first column fractions of the crude extract was run on SK-2 with the gradient elution of CHCl_3_:CH_3_OH:H_2_O from (80:20:2) to (61:32:7). SK-2/(6-12) was repeatedly applied to SK column chromatography until fraction SK-4/(1-2) was obtained. After subjecting this fraction to VLC with CH_3_OH:H_2_O (60:40) isocratic elution, we gained fraction (9-14) and applied the fraction to SPH-1. Fraction (15-20) from SPH-1 was applied on another VLC with CH_3_OH:H_2_O from (40:60) to (50:50) isocratic elution. VLC-2/(28-34) was gained as pure compound (**Compound 4**, 13.5 mg).

The identical protocol applied for compound 2 was conducted until getting fractions from VLC-10. VLC-10/(80-82) was the pure compound (**Compound 5**, 3.6 mg).

The fraction SK-1/(23-24) as one of the first column fractions of the crude extract was applied on SK-17 accompanied with CH_3_COOC_2_H_5_:CH_3_OH:H_2_O (100:17:13) isocratic elution. Fraction (6-8) from SK-17 was repeatedly applied to SPH chromatography until fraction (6-11) was obtained from SPH-26. After subjecting this fraction to VLC-13 accompanied by CH_3_OH:H_2_O gradient elution from (40:60) to (55:45), fraction (33-51) from VLC-13 was subjected to another two rounds of SK column chromatography by using gradient elution of CH_3_COOC_2_H_5_:CH_3_OH:H_2_O and CHCl_3_:CH_3_OH:H_2_O, respectively in order to isolate SK-20/(15-26) as pure compound (**Compound 6**, 13 mg).

The fraction SK-1/(23-24) as one of the first column fractions of the crude extract was applied to SK-17 accompanied by CH_3_COOC_2_H_5_:CH_3_OH:H_2_O (100:17:13) isocratic elution. Fraction (4-5) was run on SPH-23 to get fraction (18-25) which was almost pure. Following fraction (18-25) from SPH-25, the yielded fraction, was applied to preparative TLC to obtain completely pure compound (**Compound 7**, 17.8 mg).

The same protocol was conducted, as we followed for compound 7, until we yielded the fractions of SK-17. Distinct from the fraction (4-5) of SK-17 for RAT-1, we applied the fraction (2-3) of SK-17 to preparative TLC to get the pure compound (**Compound 8**, 6 mg).

Initially, the crude extract was processed by SK-1 with gradient elution from CH_2_Cl_2_: CH_3_OH (95:5) to CH_2_Cl_2_:CH_3_OH:H_2_O (60:40:4). The 33th fraction of SK-1 was applied to SK-2 accompanied by gradient elution of CHCl_3_:CH_3_OH:H_2_O from (80:20:2) to (61:32:7). SK-2/(22-24) was subjected to SK-6 with CH_3_COOC_2_H_5_:CH_3_OH:H_2_O:HCOOH (100:17:13:0.5) isocratic elution. SK-6/(2-6) was run on SK-7 accompanied with CHCl_2_:CH_3_OH:H_2_O:HCOOH gradient elution from (70:30:3:0.5) to (61:32:7:0.5). SK-7/(3-4) was applied to SPH-5 and fraction (23-49), which was almost pure, was applied to preparative TLC to obtain the completely pure compound (**Compound 9**, 13.5 mg).

The same steps performed to isolate compound 9 were conducted to obtain fraction (5-22) from SPH-5. SPH-5/(5-22) was applied to MPLC followed by CH_3_OH:H_2_O gradient elution from (45:55) to (100:0) as reverse phase chromatography. Fraction (45-80) was applied to preparative TLC and the mixture of two compounds was yielded (**Compound 10**, **compound 11**, 12 mg).

### Chemicals

To have significant amounts for mechanistic studies available, anthraquinones were purchased from commercial sources. Aloe-emodin (purity (HPLC): min. 97 area %) was obtained from TCI Deutschland GmbH (Eschborn, Germany) and was dissolved in DMSO. Emodin (purity after HPLC ≥ 90%), physcion (purity after TLC ≥ 98% TLC) and rhein (technical grade) (from Sigma, Turkey) were also dissolved in DMSO. TNF-α was obtained from Biotrend Chemikalien GmbH (Köln, Germany). Doxorubicin was provided by the University Hospital of Johannes Gutenberg University (Mainz, Germany).

### Cell culture

The cell lines used in the present work, their origins and maintenance conditions were previously reported [60–63]. In brief, drug-sensitive CCRF-CEM and multidrug resistant P-glycoprotein-over-expressing CEM/ADR5000 leukemia cells, MDA-MB-231-pcDNA3 breast cancer cells and their transfectant subline MDA-MB-231-BCRP clone 23, HCT116 (*p53*^*+/+*^) colon cancer cells and its knockout clone HCT116 (*p53*^*-/-*^), U87.MG glioblastoma multiform cells and their transfectant subline U87.MGΔEGFR as well as HEK293 human embryonic kidney cells transfected with or without a cDNA for *ABCB5* were used. The human peripheral mononuclear cells (PMNC) were isolated from fresh blood samples of a healthy donor by using Histopaque^®^ (Sigma-Aldrich, Taufkirchen, Germany).

Normal human peripheral mononuclear cells (PMNC) were isolated from fresh blood samples of a healthy donor using Histopaque^®^ (Sigma-Aldrich). In brief, 6 mL blood were layered with 6 mL Histopaque^®^ and centrifuged (400 × g) for 30 min at 4°C. The opaque interface containing lymphocytes and other mononuclear cells was transferred into a new tube and washed several times. Isolated PMNCs were kept in Panserin 413 medium (PAN-Biotech, Aidenbach, Germany) supplemented with 2 % phytohemagglutinin M (PHA-M, Life Technologies, Darmstadt, Germany).

### Resazurin reduction assay

Resazurin reduction assay was conducted to test the cytotoxicity of the compounds. This assay is based on the reduction of resazurin to resorufin by viable cells [64]. Non-viable cells do not show a blue staining because they lost their metabolic capacity which causing reduction of resazurin. Briefly, aliquots of 0.5×10^4^ adherent cells which were allowed to attach overnight and 1×10^4^ suspension cells per well were seeded in 96-well-plates with or without the addition of varying concentrations of the test substance to get a total volume of 200 μL/well. After 72 h incubation and addition of resazurin (Sigma-Aldrich) for 4 h, staining was measured by an Infinite M2000 ProTM plate reader (Tecan, Germany) using an excitation wavelength of 544 nm and an emission wavelength of 590 nm. Each assay was independently performed for at least three times, with six parallel replicates each. The protocol has been recently reported [65]. Fifty percent inhibition concentrations (IC_50_) represent the drug concentrations required to inhibit 50% of cell proliferation, which were fitted with nonlinear regression using GraphPad^®^ Prism7.

### Protease viability marker assay

The protease viability marker assay was performed to exclude the possibility that the cytotoxicity of Aloe-emodin measured by the resazurin assay was artificially influenced by any non-intended interaction with Aloe-emodin-induced ROS generation. The assay measures the protease activity within living cells. The protease activity is restricted to intact viable cells and is measured using a flourogenic, cell permeant, peptide substrate (glyxyl-phenylalanyl-aminoflourocoumarin; GF-AFC). The substrate enters intact cells, where it is cleaved by proteases to generate a fluorescent signal proportional to the number of living cells. Briefly, CCRF-CEM cells (2×10^4^cells/well) were seeded in 96 well plate. Aloe-emodin was added in a dose dependent manner (0.001 – 100 μM). After 72 h, 100 μL of GF-AFC substrate (Promega, Madison, USA) were added to each well, cells were incubated for 30 min at 37°C. Using Infinite M2000Pro™ plate reader (Tecan, Crailsheim, Germany), the fluorescent intensity was measured at an excitation wavelength 400 nm, and emitted light was collected at 505 nm.

### Cell cycle analysis

CCRF-CEM cells (2×10^4^ ) were treated with 0.5-, 1-, 2- or 4-fold IC_50_ of Aloe-emodin, respectively, for 6, 24, 48 or 72 h. The cells were collected, washed in PBS and fixed with ice-cold 96% ethanol. After washing the cells with PBS again, the cells were dissolved in PBS and stained with propidium iodide (PI, Sigma-Aldrich) at a final concentration of 50 μg/mL for 15 min in the dark. Cell cycle analyses were performed using a BD Accuri™ C6 Flow cytometer (Becton-Dickinson, Heidelberg, Germany) at 488 nm excitation wavelength, and emission was measured by a 610/20 nm band pass filter. All experiments were performed at least in triplicates. The protocol has been recently reported by us [66].

### Detection of early apoptosis and necrosis

A commercial annexin V/PI detection apoptosis kit was used to detect early apoptosis and necrosis according to the manufacturer’s instructions (Life Technologies, Carlsbad, CA, USA). Annexin V is a calcium-dependent phospholipid-binding protein, which binds to phosphatidylserine (PS). PS is predominantly located at the inner side of the plasma membrane under normal conditions and moves to outer surface of the membrane upon the onset of early apoptosis. This can be detected FITC-labeled annexin V. PI is a marker of late apoptosis and necrosis. We treated aliquots of 1×10^6^ CCRF-CEM cells with 0.5-, 1-, 2- and 4-fold IC_50_ of Aloe-emodin for 48, 72 or 96 h, respectively. After washing the cells with PBS, they were stained with annexin V/FITC at room temperature for 10-15 min. Subsequently, cells were washed again and stained with PI in the dark. Then, the results were analyzed by a BD Accuri™ C6 flow cytometer at excitation wavelength 488 nm and emission wavelength 530 nm to record annexin V/FITC signals. The fluorescence of PI was detected at 488 nm excitation and was measured by a 610/20 nm band pass filter. At least three independent experiments were performed. [65].

### Detection of reactive oxygen species (ROS)

2′,7′-Dichlorofluorescin diacetate (H_2_DCFH-DA, Sigma-Aldrich) is a cell-permeable non-fluorescent probe used to detect cellular ROS levels. In the presence of ROS, the compound is de-esterified intracellularly and converts into the highly fluorescent 2′,7′-dichlorofluorescein upon oxidation. Thus, it can be measured by flow cytometry [67]. CCRF-CEM cells were re-suspended in PBS and incubated for 30 min withH_2_DCFH-DA at a concentration of 2 μM. After washing with PBS, the cells were treated with DMSO as negative control, H_2_O_2_ and doxorubicin as positive controls or varying concentrations of Aloe-emodin (0.5-, 1-, 2- and 4-fold IC_50_) for 1 h. The results were assessed by a BD Accuri™ C6 flow cytometer (Becton Dickinson) using FL-1 the channel (488 nm excitation). For each sample, 10^4^ cells were counted. The protocol has been recently reported by us [68].

### Comet assay

The OxiSelect™ Comet Assay Kit (Cell Biolabs/Biocat, Heidelberg, Germany) was used to detect DNA damage according to the manufacturer’s instructions. CCRF-CEM cells treated with 1-, 2- and 4-fold IC_50_ of Aloe-emodin for 24 h. Then, the cells were mixed with agarose and applied to OxiSelect™ Comet Assay slides. These slides including the embedded cells were treated with lysis buffer and alkaline solution. Subsequently, electrophoresis was performed on the slide with a voltage of 22 V for 30 min corresponding to 1 V/cm of the electrophoresis chamber. These slides were washed with distilled water. After fixation with 70% ethanol, the slides were stained with by a fluorescent DNA binding dye [69].

Then, the slides were photographed by a fluorescence microscope (EVOSs FL Cell Image System, Thermo Fisher Scientific Waltham, USA) using a FITC filter with an excitation wavelength of 490 nm and emission at 520 nm. At least 50 cells per image were randomly selected and analyzed with the OpenComet software (http://www.cometbio.org). Percentages of tail DNA, tail moment and olive moment were assessed as parameters for DNA damage. The statistical significance was determined by one-way ANOVA with Tukey’s multiple comparison test.

### Measurement of mitochondrial membrane potential (MMP)

The JC-1 Mitochondrial Membrane Potential Assay Kit (Cayman Chemical, Ann Arbor, MI, USA) was applied for the detection of MMP by flow cytometry according to the manufacturer’s instructions. The cationic dye, 5, 5’, 6, 6’- tetrachloro- 1, 1’, 3, 3’- tetraethylbenzimidazolylcarbocyanine iodide (JC-1) enters the mitochondria and changes its fluorescent properties based on the aggregation of the probe. In healthy cells having high MMP, JC-1 forms complexes known as J-aggregates with intense red fluorescence. On the other hand, in cells with low MMP, JC-1 remains in its monomeric form showing green fluorescence [70]. Aliquots of 5×10^5^ cells/ml were treated with DMSO as negative control, doxorubicin as positive control or 1-, 2- or 4-fold IC_50_ of Aloe-emodin for 24 and 48 h. A LSR-Fortessa FACS analyzer (Becton–Dickinson) was used to detect the J-aggregate form of JC-1 with an excitation wavelength of 535 ± 20 nm and an emission wavelength of 590 ± 20 nm as well as the monomeric form of JC-1 at excitation and emission wavelengths of 485 and 535 nm, respectively. The results were analyzed by the FlowJo software (Celeza, Olten, Switzerland). 2×10^4^ cells were counted for each experiment which were repeated in triplicate [71].

### Microarray gene profiling

Total RNA was isolated by InviTrap Spin Universal RNA Mini kit (Stratec Molecular, Berlin, Germany) according to the manufacturer’s instructions. The quality control of total RNA, probe labeling, hybridization, scanning and data analysis was performed in the Genomics and Proteomics Core Facility at the German Cancer Research Center (DKFZ, Heidelberg, Germany). Details have been previously described [72]. The Chipster software was used to filter the set of differentially expressed genes obtained from microarray hybridization (http://chipster.csc.fi/) with a *p* value lower than 0.05. These filtered genes with fold-changes of more than 1-fold were selected for Ingenuity Pathways Analysis Software (http://www.ingenuity.com/ Ingenuity Systems, Redwood City, CA, USA) to obtain profiles of genetic networks and signaling pathways. The protocol has been recently reported by us [73].

### COMPARE and hierarchical cluster analyses

A panel of 60 cell lines from the National Cancer Institute (NCI), USA were used to perform COMPARE. Logarithmic IC_50_ values (log_10_IC_50_)have been deposited at the NCI database (http://dtp.cancer.gov/databases_tools/default.htm). The mRNA expression values of the NCI cell lines were determined via microarray analyses were deposited at the NCI website (http://dtp.cancer.gov/databasestools/default.htm) as well. These data were used to generate rank ordered lists of genes expressed in the NCI cell lines panel using COMPARE analyses [32]. Briefly, the selected genes of the NCI microarray database were ranked for similarity of its mRNA expression values to the log_10_IC_50_ values for Aloe-emodin..

Objects were categorized by determination of distances with regard to the closeness of between-individual distances to conduct hierarchical cluster analysis. All objects were assembled into dendrograms. Grouping of objects with similar properties provokes cluster formation. Distances of subordinate cluster branches to superior cluster branches serve as criteria for the closeness of clusters. Thus, objects with tightly related features were clustered closely, while separation of objects in the dendrogram increased with progressive dissimilarity. We applied the WARD method using the WinSTAT program (Kalmia, Cambridge, MA, USA). The protocol has been recently reported by us [74].

The distribution of cell lines sensitive or resistant to Aloe-emodin was calculated using the χ^2^ test. The χ^2^-test was performed to determine bivariate frequency distributions of pairs of nominal scaled variables. It was used to calculate significance values (*p*-values) and rank correlation coefficients (*R*-values) as relative measure for the linear dependency of two variables. This test was implemented into the WinSTAT program (Kalmia Co.). The χ^2^-test determines the difference between each observed and theoretical frequency for each possible outcome, squaring them, dividing each by the theoretical frequency, and taking the sum of the results. To perform the χ^2^-test, the median log_10_IC_50_ value of all cell lines tested for Aloe-emodin was used as cut-off to separate tumor cell lines as being ‘‘sensitive’’ or ‘‘resistant’’.

### Quantitative real-time polymerase chain reaction (qPCR)

Primer sequences of *HHEX*, *MCMDC2* and *CRCP* were designed using NCBI and GenScript Real Time PCR Primer Design (https://www.genscript.com/ssl-bin/app/primer) websites. *DUSP6*, *HHEX*, *MCMDC2* and *CRCP* primers were synthesized by Eurofins MWG Operon (Ebersberg, Germany) and their sequence specificities were checked by NCBI Primer-Blast (http://www.ncbi.nlm.nih.gov/tools/primer-blast). The reaction properties of primers were calculated by Eurofins genomics (https://www.eurofinsgenomics.eu/en/dna-rna-oligonucleotides/oligo-design-more/oligo-property-scan.aspx). The primer sequences were as follows: *DUSP6*, forward (5′–3′): CCTGAGGCCATTTCTTTCATAGA, reverse (5′–3′): GTCACAGTGACTGAGCGGCTAAT [75]; *HHEX*, forward: TCTACTCTGGAGCCCCTTCT, reverse: GGTTTTGACCTGTCTCTCGC; *MCMDC2*, forward: TGCGGCTTCTAGACAGTTCA, reverse: GAGCTTGTTCTGATTCTGCG; *CRCP*, forward: GGGGAGAAGAAACATGGTGA, reverse: CCGTGGAGGAAATCTTTCAA. Total RNA was isolated by InviTrap Spin Universal RNA Mini kit (Stratec Molecular) according to the manufacturer’s instruction. One microgram RNA was converted to cDNA using RevertAid H Minus First Strand cDNA Synthesis Kit (Thermo Scientific). The mRNA levels were analyzed by using of 5×Hot Start Tag EvaGreen^®^ qPCR Mix (no ROX) (Axon Labortechnik, Kaiserslautern, Germany) and CFX384^™^ Real-Time PCR Detection System (Bio-Rad, Munich, Germany). RT-PCR was performed with an initial denaturation at 95°C for 10 second followed by 40 cycles including strand separation at 95°C for 15 s, annealing at 62°C for 30 s and extension at 95°C for 1 min. The *GAPDH, HPRT1* and *RLP13* genes were used as housekeeping genes. The expression data were normalized using Bestkeeper software [76]. Among the three housekeeping genes investigated, *GAPDH* was the most stable one. The regression analyses using Bestkeeper for *GAPDH* revealed an R-value of 0.9. Therefore, *GAPDH* was used for normalization. Forward and reverse primer sequences of *GAPDH* were TGAAGGTCGGAGTCAACGGATTTGGT for forward and CATGTGGGCCATGAGGTCCACCAC for reverse, respectively [77].

Furthermore, forward and reverse primer sequences of HPRT1 were AGATGTGATGAAGGAGATGGG for forward and ACCAAGGAAAGCAAAGTCTG for reverse as well as of RLP13A were TATGCTGCCCCACAAAACC for forward and TTTCTCTTTCCTCTTCTCCTCC for reverse, respectively.

The quantification method was done as follow:
ΔCt = Ct gene test – Ct endogenous control.
ΔΔCt = ΔCt sample1 – ΔCt calibrator.
RQ = Relative quantification = 2-ΔΔCt.

The RQ is the fold-change compared to the calibrator (untreated sample, time zero, etc.). The calibrator has an RQ value of 1. All samples were compared to the calibrator.

A RQ of 10 means that this gene is 10 times more expressed in sample x than in the calibrator sample. A RQ of 0.1 means that the gene is 10 times less expressed.

## Abbreviations

DTP: Developmental Therapeutics Program
GF-AFC: glyxyl-phenylalanyl-aminoflourocoumarin
IPA: Ingenuity pathway analysis
MMP: mitochondrial membrane potential
MPLC: medium pressure liquid chromatography
NCI: National Cancer Institute
PMNC: peripheral mononuclear cells
ppm: parts per million
SPH: Sephadex column chromatography
SK: silica gel column chromatography
qPCR: quantitative real-time polymerase chain reaction
ROS: reactive oxygen species
TLC: thin layer chromoatography
VLC: vacuum liquid chromatography
