# Correction: Aloeemodin as drug candidate for cancer therapy

**DOI:** 10.18632/oncotarget.28870

**Published:** 2026-04-24

**Authors:** Nadire Özenver, Mohamed Saeed, Lütfiye Ömur Demirezer, Thomas Efferth

**Affiliations:** ^1^Department of Pharmacognosy, Faculty of Pharmacy, Hacettepe University, 06100 Ankara, Turkey; ^2^Department of Pharmaceutical Biology, Institute of Pharmacy and Biochemistry, Johannes Gutenberg University, 55128 Mainz, Germany

**This article has been corrected:** In Figure 9A, the flow cytometry plot for 4 × IC50 was unintentionally repeated instead of the 2 × IC50 plot. This has been corrected using the original data.

Additionally, the authors identified two errors in Figure 9C. First, the column graph for the 0.1 µM doxorubicin concentration was found to be inconsistent with its corresponding flow cytometry plot. Second, the column graph for the 1 µM doxorubicin concentration incorrectly indicated a percentage of AV-/PI- cell population of 1% instead of the 0.1% shown in the flow cytometry plot. Both graphs in Figure 9C have now been corrected to accurately reflect the relevant flow cytometry data. These corrections do not change the conclusions of the paper. The authors apologize for any inconvenience caused.

The corrected Figure 9 is presented below.

Original article: Oncotarget. 2018; 9:17770–17796. 17770-17796. https://doi.org/10.18632/oncotarget.24880

**Figure 9:**
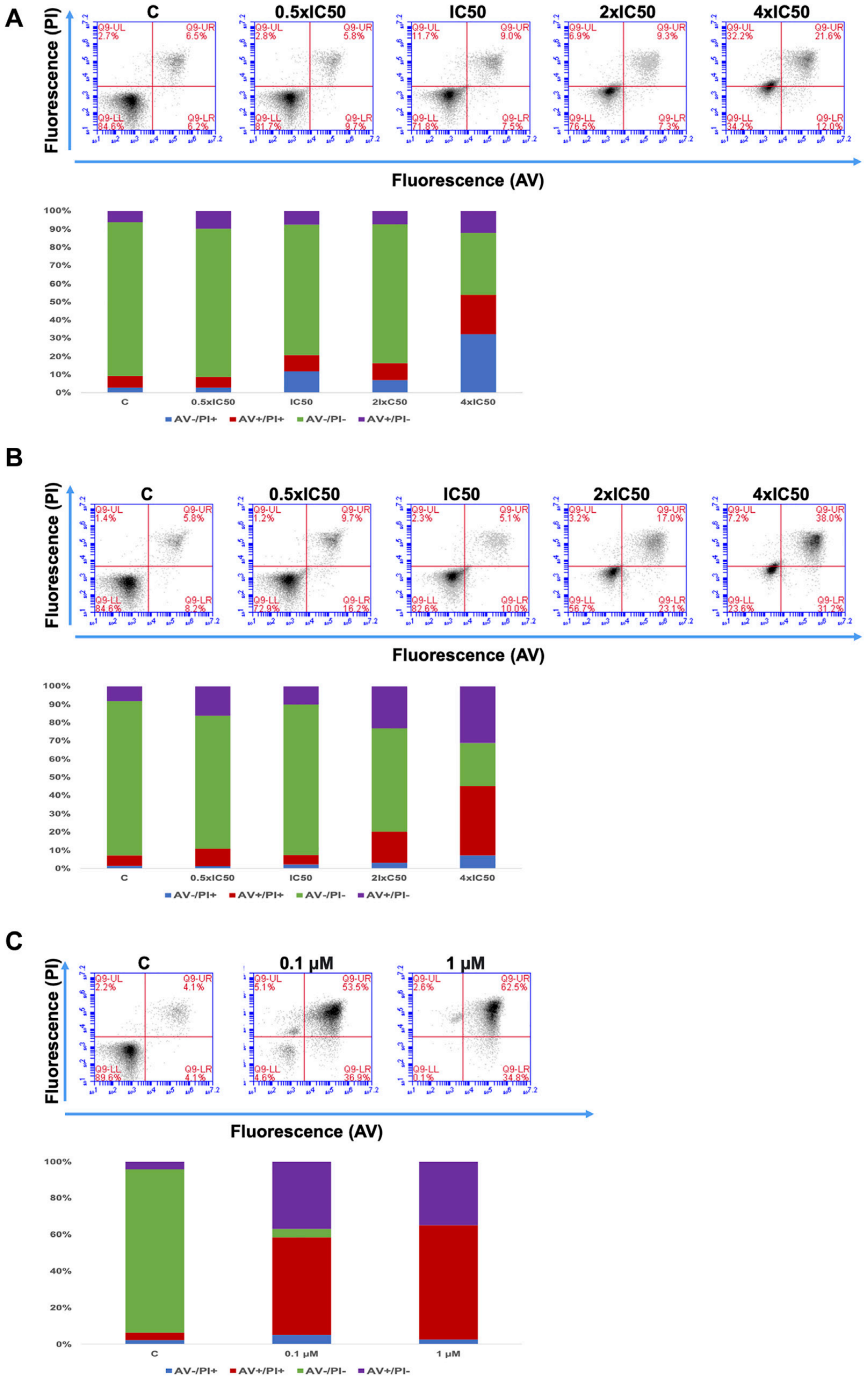
Apoptosis effect in CCRF-CEM cells of Aloe-emodin for 72 h (**A**) and 96 h (**B**) and of doxorubicin for 72 h (**C**).

